# Integrative overview of the herpetofauna from Serra da Mocidade, a granitic mountain range in northern Brazil

**DOI:** 10.3897/zookeys.715.20288

**Published:** 2017-11-22

**Authors:** Leandro J.C.L. Moraes, Alexandre P. de Almeida, Rafael de Fraga, Rommel R. Rojas, Renata M. Pirani, Ariane A.A. Silva, Vinícius T. de Carvalho, Marcelo Gordo, Fernanda P. Werneck

**Affiliations:** 1 Coordenação de Biodiversidade, Instituto Nacional de Pesquisas da Amazônia, Av. André Araújo 2936, 69067-375, Manaus, Amazonas, Brazil; 2 Departamento de Biologia, Universidade Federal do Amazonas, Av. General Rodrigo Octávio Jordão Ramos 3000, 69077-000, Manaus, Amazonas, Brazil

**Keywords:** Amazonia, bioacoustics, biogeography, lowland forest, morphology, mountain, mtDNA, Pantepui, phylogenetic relationships

## Abstract

The Brazilian mountain ranges from the Guiana Shield highlands are largely unexplored, with an understudied herpetofauna. Here the amphibian and reptile species diversity of the remote Serra da Mocidade mountain range, located in extreme northern Brazil, is reported upon, and biogeographical affinities and taxonomic highlights are discussed. A 22-days expedition to this mountain range was undertaken during which specimens were sampled at four distinct altitudinal levels (600, 960, 1,060 and 1,365 m above sea level) using six complementary methods. Specimens were identified through an integrated approach that considered morphological, bioacoustical, and molecular analyses. Fifty-one species (23 amphibians and 28 reptiles) were found, a comparable richness to other mountain ranges in the region. The recorded assemblage showed a mixed compositional influence from assemblages typical of other mountain ranges and lowland forest habitats in the region. Most of the taxa occupying the Serra da Mocidade mountain range are typical of the Guiana Shield or widely distributed in the Amazon. Extensions of known distribution ranges and candidate undescribed taxa are also recorded. This is the first herpetofaunal expedition that accessed the higher altitudinal levels of this mountain range, contributing to the basic knowledge of these groups in remote areas.

## Introduction

Scientific interest in mountain ranges arises primarily because they are characterized by a geographical isolation associated with differential availability of topographical, climatic, and edaphic conditions along the altitudinal gradients (Haslett 1997, Martinelli 2007, Körner et al. 2017). These characteristics provide ideal conditions for the development of unique evolutionary lineages and occurrence of a variety of endemic taxa (Lomolino 2001, Rull 2005, Hoorn et al. 2013, Nogué et al. 2013). This speciation hotspot pattern occurs in the mountain ranges of northern South America (Guiana Shield highlands), which lies on the ancient terrain of the Cratonic Guiana Shield (Hershkovitz 1969, Désamoré et al. 2014, Salerno et al. 2012, Bonaccorso and Guayasamin 2013). Despite a history of different concepts and geographic boundaries (Mayr and Phelps 1967, Huber 1988a, Kok 2013a), these high altitude areas are considered as a distinct biogeographic region (Morrone 2014), presenting biotic affinities with the megadiverse Amazon and Andean regions (Duellman 1979, Salerno et al. 2012, Mannion et al. 2014).

Initial discoveries concerning the amphibian and reptile diversity from Guiana Shield highlands were reported by localized expeditions at the transition between the nineteenth and twentieth centuries (Boulenger 1900, Roze 1958a, b). Knowledge increased exponentially when helicopters facilitated access to remote mountains (Aubrecht et al. 2012), leading to several expeditions focused on describing the assemblages of particular localities. Such studies brought to prominence several endemic taxa from the highlands (e.g., Gorzula 1992, Myers 1997, Myers and Donnelly 1992, 1996, 2001, 2008, MacCulloch et al. 2007, Barrio-Amorós and Brewer-Carias 2008, Kok 2008, 2009a, 2009b, 2010, 2013b, 2015, Kok and Rivas 2011, Kok et al. 2010, 2011, 2015), and some resulted in broad outlines of the main biogeographical patterns of these taxa (e.g., Hoogmoed 1979, Duellman 1999, Gorzula and Señaris 1999, McDiarmid and Donnelly 2005).

More recently, studies have shown that diversification and evolutionary patterns of distinct species were associated with the landscape history of the region (Kok et al. 2012, 2017, Salerno et al. 2012, 2015, Vacher et al. 2017). While several intriguing patterns have been found for some highland lineages, such as recent diversification and low genetic divergence among mountains (Salerno et al. 2012, Kok et al. 2012, 2017), overall knowledge on the geographical and altitudinal distribution patterns of amphibians and reptiles from Guiana Shield highlands and their drivers are far from being fully understood. One of the main reasons is the occurrence of huge sampling gaps, mostly due to the short-term nature of inventories and to the difficult and costly access to highland areas, some of which remain unexplored (Aubrecht et al. 2012). The sampling deficiency in the Brazilian region of the Guiana Shield highlands highlights the importance of exploring these areas for biodiversity and biogeography assessments. Sampling in novel mountain ranges will most likely result in the discovery of new taxa and unique lineages, and geographical range extensions, all of which can contribute to the conservation of these threatened regions (Rull et al. 2016).

Furthermore, most specimens currently collected during biological inventories are identified using morphological characteristics. However, given the pervasive occurrence of cryptic diversity (Vences et al. 2005, Vences and Wake 2007), particularly in Neotropical amphibians and reptiles (Fouquet et al. 2007a, c, Geurgas and Rodrigues 2010, Oliveira et al. 2016, Kok et al. 2016), other methods are being used to reveal the hidden diversity in remote areas with difficult access. For example, molecular techniques may contribute to indicate the presence of undescribed species and detect cryptic speciation through divergence in DNA sequences, and this short-term result might take longer to be achieved using a single taxonomic data source (Vences et al. 2005, Vences and Wake 2007, Fouquet et al. 2007a, Paz and Crawford 2012).

Recently, a multidisciplinary initiative conducted an expedition (“Biodiversity of the Serra da Mocidade”) to inventory the biological diversity of distinct taxonomic groups in the poorly known region of the Serra da Mocidade, a remote granitic mountain range located in northern Brazil (INPA, 2016), highly isolated from other mountains and with difficult access. Here we present and discuss the diversity of amphibians and reptiles of the area and their biotic affinities, using an integrative approach combining morphological, bioacoustical and molecular analyses to identify specimens. We found remarkable records, and make observations concerning species taxonomy, ecology and distribution patterns in the Guiana Shield highlands region.

## Materials and methods

### Study area

**Figure 1.**
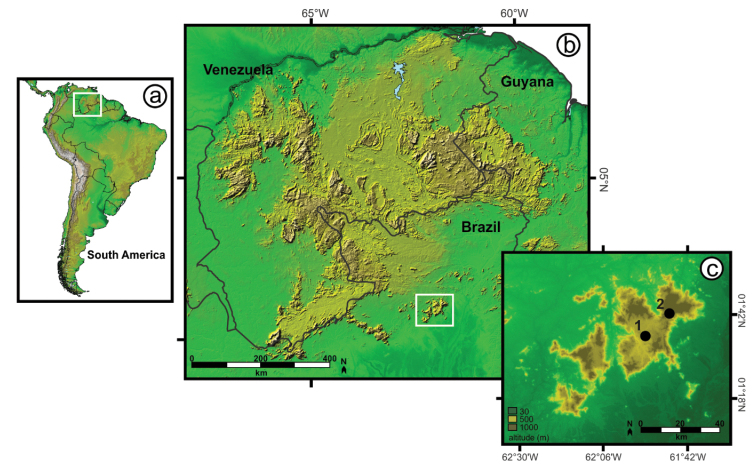
Study area. Location of **a** the main Guiana Shield highlands region in northern South America, and **b** the Serra da Mocidade mountain range. A larger scale map of mountain range **c** shows the location of the two field base camps.

The Guiana Shield highlands region is located in northern South America, within the limits of Venezuela, Guyana, Suriname, and Brazil (Fig. 1), and is composed of mountain ranges covered by dense forests, shrubby vegetation or moss forests, surrounded by a lowland matrix of either tropical forests or savanna ecosystems (Mayr and Phelps 1967, Huber 1998b, McDiarmid and Donnelly 2005). These ancient mountains date to the Precambrian period (1.8–2.5 billion years) (Santos et al. 2003, Nogué et al. 2009, Kok 2013a), and have two main geological origins: sedimentary rocks, which are currently exposed as abrupt vertical elevations with tabular tops (called “tepuis”) and igneous-metamorphic rocks, which lie beneath the sedimentary rocks and may also be exposed as granitic uplifts with a mountain-like appearance (Steyermark 1986, Schubert and Briceño 1987, Huber 1995, Hoorn and Wesselingh 2010).

**Figure 2.**
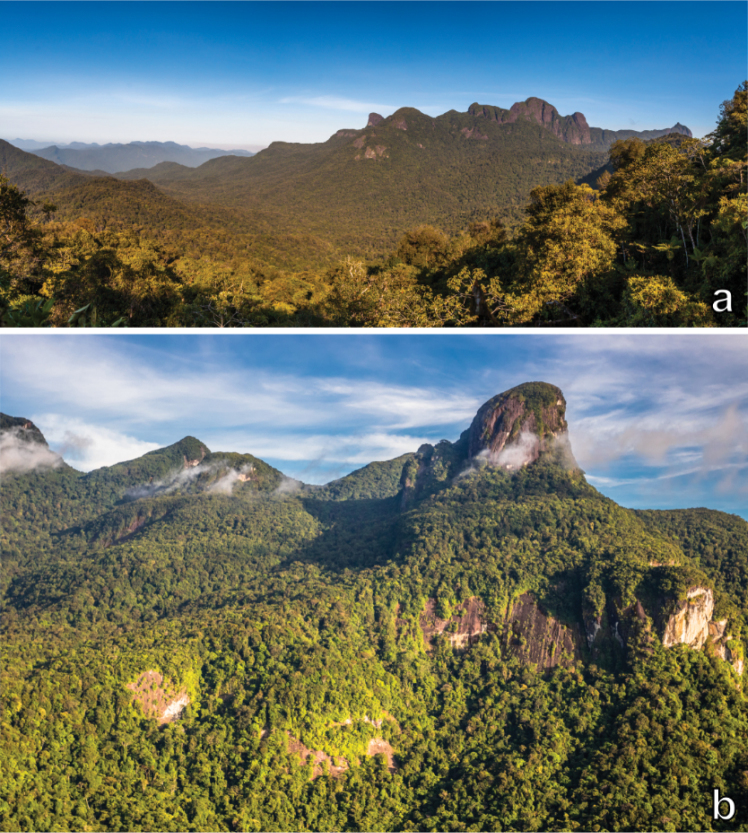
Serra da Mocidade. Panoramic views of the Serra da Mocidade mountain range (**a**) and a typical granitic inselberg formation (**b**), covered by montane forest. Photographs by Thiago Laranjeiras.

This study was conducted at Serra da Mocidade (Figs 1, 2), a complex of granitic mountains located in extreme northern Brazil, within the limits of Caracaraí municipality, Roraima state, with a mean altitudinal level of 1,000 m above sea level (hereafter, asl) and peaks over 1,900 m asl. The Serra da Mocidade area is protected by a federal conservation unit (Parque Nacional da Serra da Mocidade), an indigenous Yanomami territory and a military area, property of the Brazilian Army (Ferreira et al. 2014, Ministério do Meio Ambiente 2016). This mountain range is isolated in relation to other Guiana Shield highlands mountains, with the closest mountain range (Serra do Aracá) ca. 100 km distant to the west.

The lowland regional climate has low annual temperature variation, ranging from 24° C to 27° C (average 26° C), a rainy season from April to September and a dry season from October to March (Alvares et al. 2013). Annual rainfall exceeds 2,500 mm (Sombroek 2001, Alvares et al. 2013). Daily thermal oscillation increases at higher altitudes, where temperatures are lower and the influence of wind is stronger, preventing establishment of taller forests (Haslett 1997). The base and lower slopes of the mountain range are covered by dense tropical submontane and montane rainforests, while increased humidity at higher altitudes fosters the development of extensive epiphyte and moss coverage on tree trunks (Ministério do Meio Ambiente 2016).

### Sampling areas and species survey

Logistic support from the Brazilian Army allowed aerial access at altitudes only accessible by helicopters, and the installation of two base camps from which it was possible to reach different altitudinal levels (Fig. 1). At base camp #1 at 600 m asl (01°36'N, 61°54'W), we sampled areas in the foothills of the mountains, while base camp #2 at 1,060 m (01°42'N, 61°47'W) allowed access to altitudinal levels of 960 m asl (01°42'N, 61°48'W) and 1,365 m asl (01°43'N, 61°45'W). Altitudinal levels closer to the camp (900–1,100 m asl) were sampled more intensively due to difficult access in the areas located at 1,365 m asl, which we sampled during a single operation. Although herpetofaunal rapid inventories already have been conducted in the lowlands of this region (Ministério do Meio Ambiente 2016), this is the first expedition that reached the higher altitudes of this mountain range.

Surveys were conducted in two teams, each with three trained herpetologists, one from each base camp (first team at base camp #1 for 15 to 23 January 2016, and second team at base camp #2 for 25 January to 06 February 2016), totaling 22 days of field work and 66 man-days of sampling effort. To maximize characterization of the regional herpetofaunal diversity we used six complementary survey methods to detect individuals: (1) active surveys (Heyer et al. 1994) conducted during day and night, where individuals were visually detected or heard in as many microhabitat as possible and manually captured; (2) pitfall traps (Campbell and Christman 1982, Heyer et al. 1994), installed in two sampling lines, each of ten 60-liters buckets spaced every 10 meters (total sampling line length 100 m), and buried in the ground with the opening at the surface level, interleaved with a pole-supported plastic fence, the lower part of which was buried in the ground; (3) trammel nets, which were installed in streams near the base camps, in order to catch turtles, aquatic amphibians and squamates (Campbell and Christman 1982); (4) hook with meat bait, specifically for turtles; (5) glue traps, installed in fallen logs and tree trunks aiming capture of arboreal squamates and (6) shotgun. All traps were visited every 24 hours or less throughout the sampling period at each base camp. Specimens obtained using methods other than the ones cited above were considered as occasional encounters (Martins and Oliveira 1988, Heyer et al. 1994).

### Collection and species identification

Specimens were killed with an injection of Thiopental® or Lidocaine, fixed with 10% formalin, and preserved in 70% ethanol. We removed muscle or liver tissue from specimens before the fixation process and stored it in absolute ethanol. Voucher specimens and tissue samples were deposited in the Collections of Amphibians and Reptiles (INPA-H) and of Genetics Resources (INPA-HT) of the Instituto Nacional de Pesquisas da Amazônia (INPA), Manaus, Amazonas, Brazil, respectively.

Taxonomic identifications were performed using morphological, bioacoustical, and molecular analyses, following the taxonomic arrangements of Frost (2017) for amphibians and Uetz and Hošek (2017) for reptiles, with modifications by Pyron et al. (2013), Pinto-Sánchez et al. (2015), and Karin et al. (2016).

### Morphology

The specimens’ morphologies were analysed according to original descriptions, taxonomic and phylogenetic revisions, dichotomous keys, field guides and results from similar expeditions from Guiana Shield highlands (Boulenger 1900, Boulenger 1911, Roze 1958a, 1958b, 1961, 1987, Rivero 1961, 1970, Vial and Jimenez-Porras 1967, Heyer 1970, 1994, Lutz 1973, Duellman 1979, 1999, Hoogmoed 1979, 1990, Peters and Donoso-Barros 1986, Rebouças-Spieker and Vanzolini 1990, Donnelly and Myers 1991, Gorzula 1992, Dixon et al. 1993, O’Shea and Stimson 1993, Ávila-Pires 1995, Myers 1997, Myers and Donnelly 1996, 1997, 2001, 2008, Gorzula and Señaris 1999, Lescure and Marty 2000, MacCulloch and Lathrop 2002, 2009, Hollowell and Reynolds 2005, McDiarmid and Donnelly 2005, Miralles et al. 2005, Señaris and Ayarzagüena 2005, Barrio-Amorós and Molina 2006, Bergmann and Russell 2007, Fouquet et al. 2007a, 2007b, 2014, 2015a, 2015b, Hawkins et al. 2007, MacCulloch et al. 2007, Barrio-Amorós and Brewer-Carias 2008, Guayasamin et al. 2008, 2009, Harvey 2008, Kok and Castroviejo-Fisher 2008, Vogt 2008, Lima and Prudente 2009, Ávila-Pires et al. 2010, Kok 2010, 2013a Miralles and Carranza 2010, Castroviejo-Fisher et al. 2011, Maciel and Hoogmoed 2011, Hedges and Conn 2012, Mendes-Pinto et al. 2012, Motta et al. 2012, Rivas et al. 2012, Cisneros-Heredia 2013, Cole et al. 2013, Jungfer et al. 2013, Lavilla et al. 2013, 2017, Murphy and Jowers 2013, Passos et al. 2013, Gehara et al. 2014, Sá et al. 2014, van Dijk et al. 2014, Wallach et al. 2014, Feitosa et al. 2015, dos Santos et al. 2015, Ribeiro-Júnior 2015a, 2015b, Dewynter et al. 2016a, 2016b, 2016c, 2016d, Oliveira et al. 2016, Ribeiro-Júnior and Amaral 2016, 2017, Orrico et al. 2017, Kok et al. 2017, Vacher et al. 2017), as well as through comparisons with other voucher specimens deposited at INPA-H collection. We investigated external meristic, morphometric and colouration characters. For colour in life we used photographs taken during the expedition, and for morphometric comparisons we measured: the snout-vent length (SVL) of amphibians, lizards, snakes and crocodilians, the caudal length of snakes (CL), and carapace length (CAL) and width (CW) of chelonians. Specimens examined are listed in Suppl. material 1.

### Bioacoustics

The calls from some amphibian species were recorded in uncompressed wav format, with a Zoom H1 Handy Recorder (Zoom Corporations, Tokyo, Japan) equipped with an internal microphone, distant about 1–2 m to the emitter. The calls were digitised and analysed using Raven Pro. 1.5 (Cornell Laboratory of Ornithology) at a sampling frequency of 44 KHz and 16-bit resolution. Call structures were visually analysed in the spectrograms, and we measured the following quantitative parameters, considered in amphibian taxonomy (Köhler et al. 2017): call duration (s), inter-call interval (s), pulse duration (s), pulse rate (pulses/s) and dominant frequency (kHz), summarized in mean values ± standard deviation. The data were compared to calls described in the literature for each analysed species (Cardoso and Haddad 1984, Hoogmoed 1990, Donnelly and Myers 1991, Fouquet et al. 2007b, Morais et al. 2012, Fouquet et al. 2015).

### Molecular analyses for uncertain species

When specimens were part of groups already considered as a complex of multiple species or when we considered the possibility of an undescribed taxon, we explored their taxonomic status using DNA sequences of mitochondrial gene 16S, a standard marker for amphibians and reptiles (Vences et al. 2012). Genomic DNA was isolated from collected tissues using a commercial kit (Wizard, Promega Corp., Madison, WI), and target region amplified via the Polymerase Chain Reaction (PCR) using primers 16Sar and 16Sbr (Palumbi et al. 1991). Purified PCR products were sequenced using the Big Dye Terminator sequencing kit (Applied Biosystems, Waltham, USA) in automated sequencer ABI 3130 XL (Applied Biosystems, Waltham, USA) at Thematic Laboratory of Molecular Biology at INPA.

DNA sequences obtained were compared with those available for closely related taxa in GenBank (Benson et al. 2014). Sequences were manually edited and aligned with CLUSTAL X algorithm run on MEGA 6.06 software (Tamura et al. 2013). Using the same software, we generated maximum likelihood (ML) phylogenetic trees for each taxonomic group inferred by 5,000 bootstrap replicates, and estimated genetic distances between main lineages of each taxon using uncorrected pairwise distances. Although the threshold of the genetic distance percentage that represents an interspecific variation is arbitrary and varies according to the group diversification, we followed Fouquet et al. (2007a, c) and considered the possibility of new taxa when genetic distances were above 3%. In these cases, the final definition of the taxonomic status of a given specimen was thus the result of an integrated interpretation of morphological, bioacoustical, and molecular results.

### Diversity and biogeographical comparisons

In order to investigate the relationship between the recorded diversity and sampling effort, as well as to identify differences in species richness between altitudinal levels, we performed extrapolated rarefaction curves (Chao et al. 2014) with presence-absence data, considering the total sampling and at distinct altitudinal levels separately (600 and >900 m asl). We also compile results of herpetofaunal inventories conducted in main nearby habitats to compare the species richness and composition and detect the faunistic affinities of the Serra da Mocidade diversity. These habitats include several mountain ranges part of Guiana Shield highlands, with over five known species (data compiled from Boulenger 1895a, 1895b, 1900, Burt and Burt 1931, Roze 1958a, 1958b, 1987, Rivero 1961, 1966, Lancini 1968, Ayarzagüena 1983, Duellman and Hoogmoed 1984, Zweifel 1986, McDiarmid and Paolillo 1988, Robinson 1989 Mägdefrau et al. 1991, Donnelly and Myers 1991, Ayarzagüena et al. 1992, Donnelly et al. 1992, Gorzula 1992, Ayarzagüena and Señaris 1993, Señaris and Ayarzagüena 1993, Myers et al. 1993, Señaris et al. 1994, Williams et al. 1996, Myers 1997, Myers and Donnelly 1997, 2001, 2008, Fuentes and Barrio-Amorós 2004, McDiarmid and Donnelly 2005, Ouboter et al. 2007, Watling and Ngadino 2007, Barrio-Amorós and Brewer-Carias 2008, Barrio-Amorós and Duellman 2009, Carvalho et al. 2010, Castroviejo-Fisher et al. 2011, Ouboter and Jairam 2012, Fouquet et al. 2015a, Fraga et al. 2017, Rojas-Runjaic et al. 2017, INPA-H voucher specimens), as well as lowland forests and open habitats (*savana*, *campina* and anthropized areas) in northern South America, with focus on the Brazilian territory (data compiled from O’Shea 1989, 1998, Martins 1998, MacCulloch and Reynolds 2013, IBAMA 2014, ICMBio 2014, Gordo et al. 2014, Señaris et al. 2014, Silva 2016, INPA-H voucher specimens). The similarity in species composition between Serra da Mocidade and these habitats was graphically investigated through multivariate ordination using a non-metric multidimensional scaling (NMDS) (Clarke 1993), with qualitative data and the Jaccard index as a similarity measure. Rarefaction curves and NMDS ordination were generated using the R statistical software packages iNEXT (Chao et al. 2014) and vegan (Oksanen et al. 2017), respectively.

To identify biogeographical patterns and evaluate the contribution of adjacent regions to the composition of the local herpetofauna at Serra da Mocidade, we classified species according to their known geographical distribution, delimiting boundaries of biogeographic regions based on the main geological compartments for Northern South America (Gibbs and Barron 1993, Aleixo and Rossetti 2007, Hoorn and Wesselingh 2010), already known to influence the biotic distribution (Ávila-Pires et al. 1995, Aleixo and Rossetti 2007). Therefore, the species were classified in the following categories, which decrease in geographical scale: Widely distributed in Amazonia (WD), for species with wide geographical ranges throughout this region; Western Amazonia (WA) and Eastern Amazonia (EA) for species typical of these macro-regions, influenced by sedimentary basin of the Amazonas River and crystalline shields, respectively; Andes (AN) for species typical of this mountain range of western South America; Guiana Shield (GS), for species occurring in the lowlands of crystalline basement north of the Amazonas River; Guiana Shield highlands (GH), for species occurring mainly in the uplands of this mountainous complex; Potentially endemic to the Serra da Mocidade region (PE), for species potentially restricted to the studied mountain range. Finally, we also considered species that occur at punctual restricted localities outside of their main geographical range (PR) and at transition zones between Amazonia and other biomes (TZ). To evaluate the contribution of these regions in shaping the local herpetofauna, we calculated a relative percentage, dividing the number of species from each biogeographical region in relation to the total number of recorded species, for both amphibians and reptiles.

To verify the contribution of altitudinal generalists and specialists to the species composition of the Serra da Mocidade herpetofauna, we also classified species according to their known altitudinal range as reported in the literature, as lowland (occuring mainly below 500 m asl) and upland (occuring mainly above 500 m asl) species (Hoogmoed 1979, McDiarmid and Donnelly 2005). The relative contribution of these assemblages was also evaluated by dividing the number of species from each group in relation to the total number of recorded species, for both amphibians and reptiles, considering the total sampling and samples from distinct altitudinal levels.

## Results

**Figure 3.**
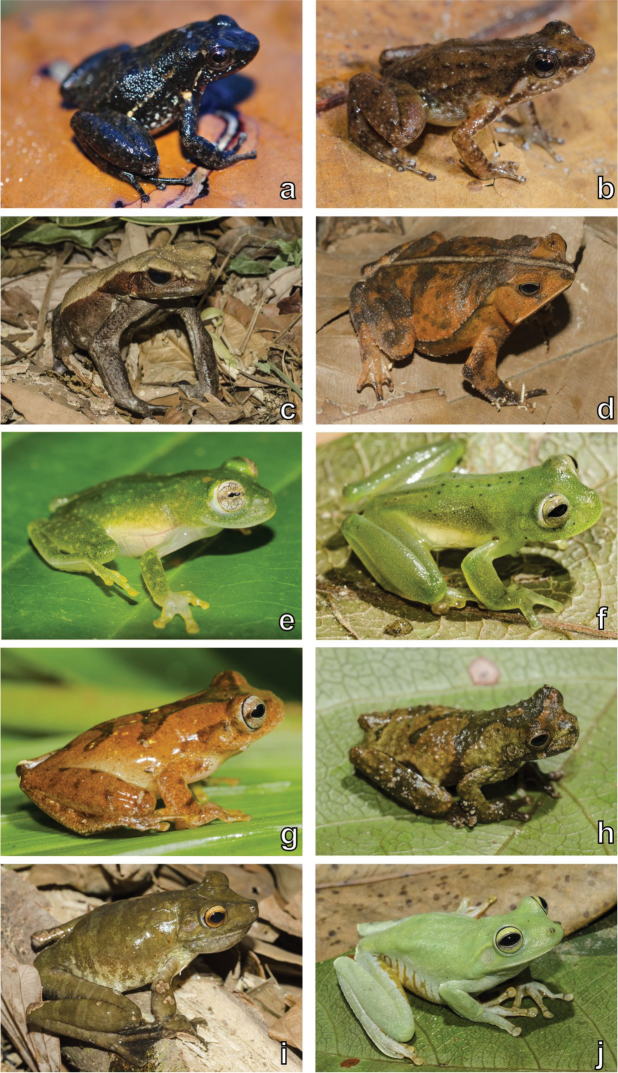
Amphibian diversity. Examples of amphibians recorded in the Serra da Mocidade mountain range. **a** *Anomaloglossus apiau*, male **b** *Anomaloglossus apiau*, female **c** *Rhaebo guttatus* **d** *Rhinella martyi* **e** Hyalinobatrachium aff. taylori **f** *Vitreorana ritae* **g** *Dendropsophus minutus* **h** *Dendropsophus parviceps* **i** *Boana boans* **j** *Boana xerophylla*. Photographs by Haroldo Palo Jr. (**c–j**).

**Figure 4.**
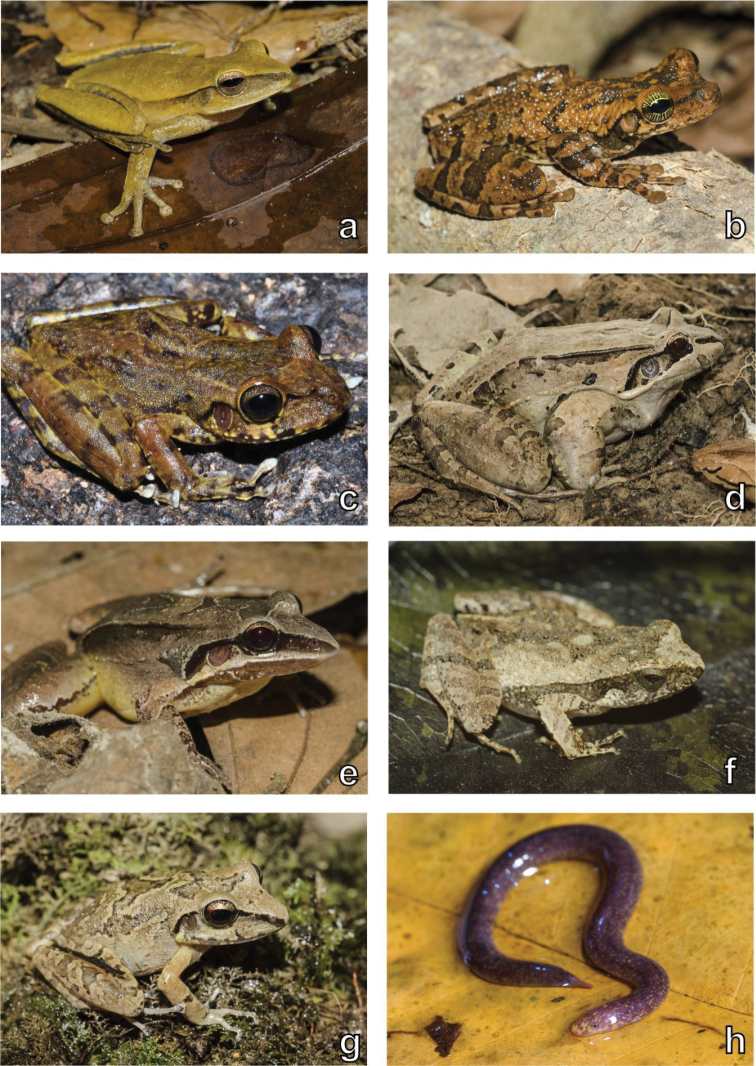
Amphibian diversity. Examples of amphibians recorded in the Serra da Mocidade mountain range. **a** *Boana multifasciata* **b** *Osteocephalus taurinus* **c** *Stefania* sp. **d** *Leptodactylus guianensis* **e** *Leptodactylus mystaceus* **f** *Physalaemus ephippifer* **g** Pristimantis aff. vilarsi **h** *Epicrionops* sp. Photographs by Haroldo Palo Jr. (**a, b, d–g**), and Marcos Amend (**h**).

**Figure 5.**
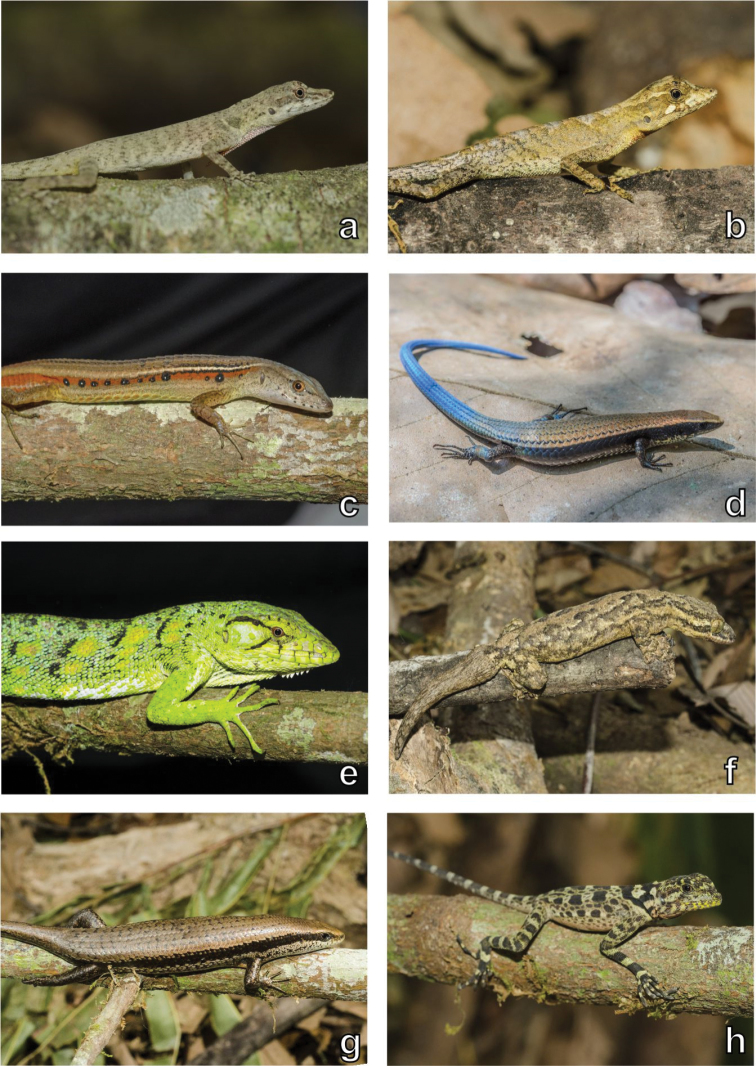
Lizard diversity. Examples of lizards recorded in the Serra da Mocidade mountain range. **a** *Anolis fuscoauratus* **b** *Anolis planiceps* **c** *Cercosaura ocellata* **d** *Tretioscincus oriximinensis* **e** *Polychrus marmoratus* **f** *Thecadactylus rapicauda* **g** *Mabuya nigropunctata* **h** *Plica plica.* Photographs by Haroldo Palo Jr. (**a–c, e–h**) and Marcos Amend (**d**).

**Figure 6.**
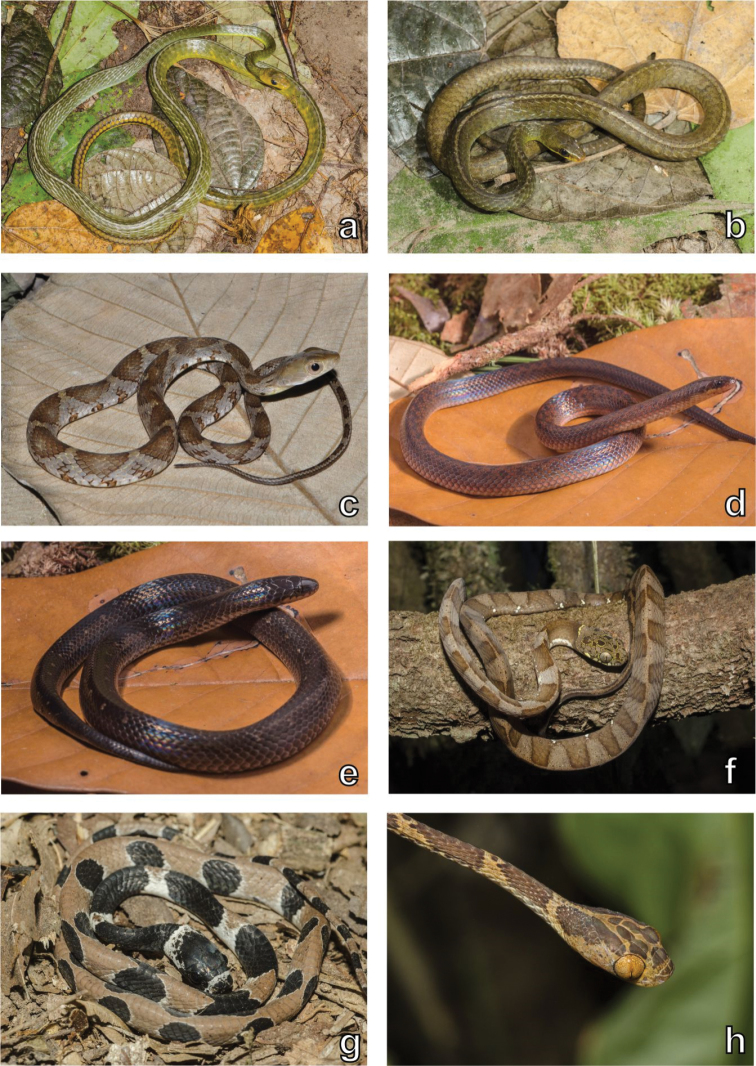
Snake diversity. Examples of snakes recorded in the Serra da Mocidade mountain range. **a** *Chironius septentrionalis* **b** *Chironius fuscus* **c** *Drymobius rhombifer* **d** *Atractus riveroi*, morph 1 **e** *Atractus riveroi*, morph 2 **f** *Dipsas indica indica* **g** *Dipsas pavonina* **h** *Imantodes cenchoa*. Photographs by Haroldo Palo Jr. (**a, b, f–h**) and Marcos Amend (**d, e**).

**Figure 7.**
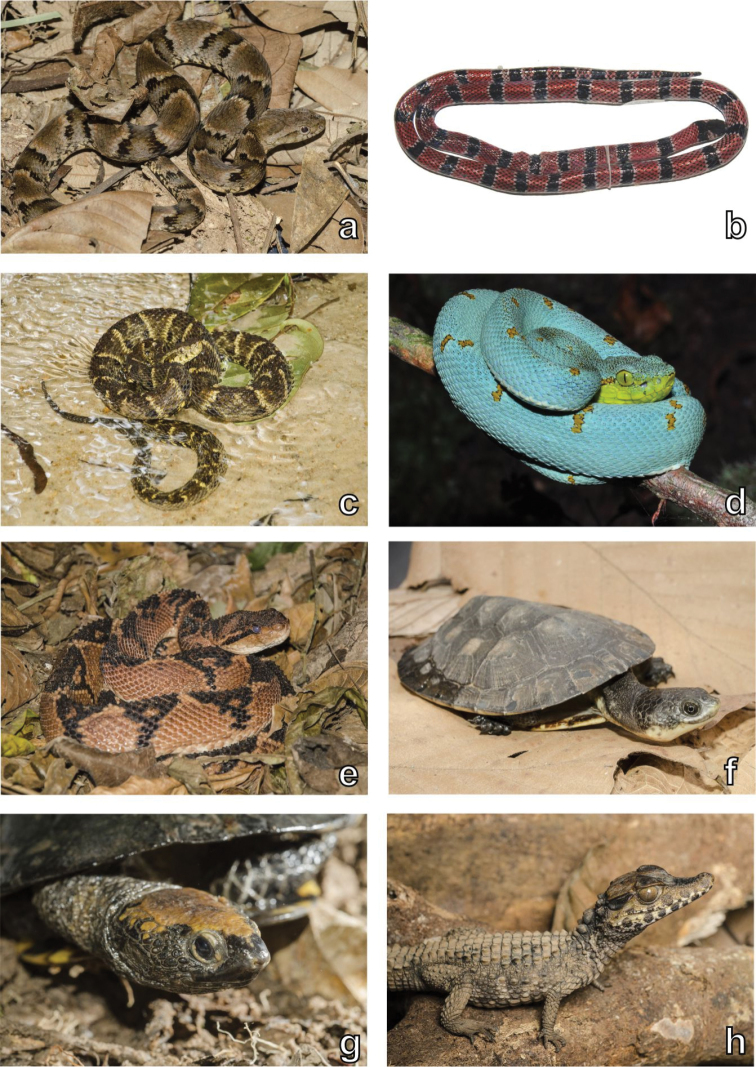
Snake, chelonian and crocodilian diversity. Examples of snakes, chelonians and the crocodilian recorded in the Serra da Mocidade mountain range. **a** *Xenodon rabdocephalus rabdocephalus* **b** *Micrurus remotus* **c** *Bothrops atrox* **d** *Bothrops bilineatus bilineatus* **e** *Lachesis muta muta* **f** *Mesoclemmys gibba* **g** *Platemys platycephala melanonota* **h** *Paleosuchus trigonatus*. Photographs by Haroldo Palo Jr. (**a, c, e–h**).

**Figure 8.**
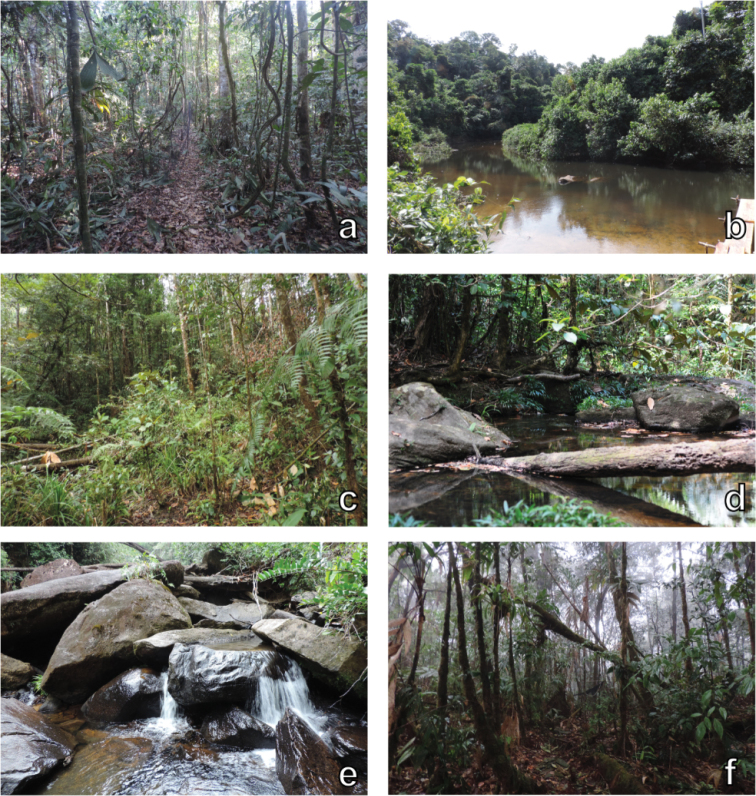
Habitat diversity. Examples of habitats sampled in the Serra da Mocidade mountain range. **a** Submontane rainforest at 600 m asl **b** Pacú River, main water body near camp #1 **c** Montane rainforest at 1,060 m asl **d, e** Rocky streams at 1,060 m asl **f** Montane rainforest at 1,365 m asl. Photographs by Ramiro Melinski (**a–c, e–f**).

**Figure 9.**
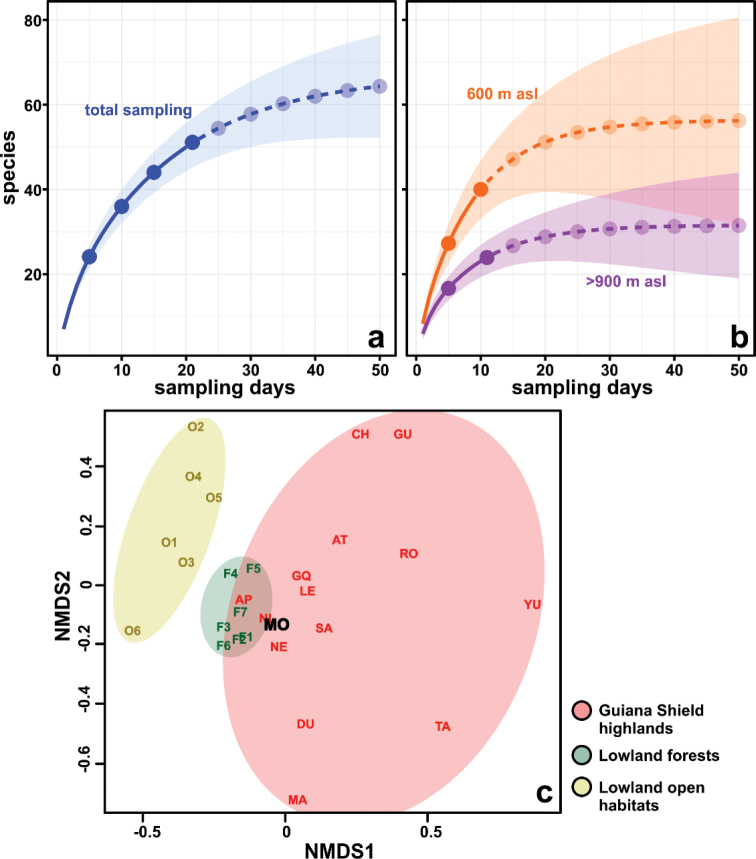
Variation in species richness and composition. **a, b** Extrapolated rarefaction curves showing variation in species richness related to sampling effort at our total sampling **a** and at distinct altitudinal levels **b** of the Serra da Mocidade mountain range. Observed data are in full lines and extrapolated in dashed lines **c** Non-metric multidimensional scaling (NMDS) ordination for amphibian and reptile assemblages from mountain ranges of Guiana Shield highlands and nearby lowland habitats, showing the mixed influence in the Serra da Mocidade composition. Mountain ranges: (MO) Mocidade; (AP) Apiaú; (Du) Duida; (Ma) Marahuaka; (AT) Auyantepui; (Ch) Chimantá; (Gq) Guaiquinima; (NE) Neblina; (TA) Tamacuari; (YU) Yutajé-Corocoro; (RO) Roraima; (SA) Sarisariñama; (NL) Nassau Lely. Lowland habitats: (F1, O1) ESEC Maracá; (F2, O2) PARNA Viruá; (F3, O3) Roraima Lowlands; (F4, O4) Samã and Miang rivers region; (F5, O5) Parque Nacional Canaima; (F6, O6) Parque Nacional da Serra da Mocidade and Estação Ecológica Niquiá; (F7) Kurupukari. References are detailed in the text.

A total of 305 specimens (232 amphibians, 58 squamates, 13 chelonians, and two crocodilians) was recorded, belonging to 51 species (23 amphibians, 24 squamates, three chelonians, and one crocodilian) from 25 families (Table 1). The most diverse families were Hylidae for amphibians (six species), Dactyloidae for lizards (three species), Colubridae for snakes (six species), and Chelidae for chelonians (two species). Some examples of this diversity and the sampled habitats appear in Figs 3–8. As expected, the species rarefaction curves show that the Serra da Mocidade mountain range still has potential to harbour a greater amphibian and reptile diversity than we record in this short-term sampling (Fig. 9a). Furthermore, the species richness differs between sampled altitudinal levels, tending to decrease in the higher altitudes (above 900 m asl) (Fig. 9b).

**Table 1.** Recorded species. List of amphibians and reptiles recorded at the Serra da Mocidade mountain range, with respective sample sizes at each distinct altitudinal level (m above sea level), sampling methods, morphological data, and species’ geographical and altitudinal distributions. Sampling methods: (AS) Active survey; (PT) Pitfall traps; (TN) Trammel nets; (HM) Hook with meat bait; (GT) Glue traps; (SG) Shotgun; (OE) Occasional encounters. Morphological measurements: (SVL) Snout-vent length; (CL) Caudal length; (CAL) Carapace length; (CW) Carapace width. Geographical distribution: (WD) Widely distributed in Amazonia; (WA) Western Amazonia; (EA) Eastern Amazonia; (AN) Andes; (GS) Guiana Shield; (GH) Guiana Shield highlands; (PE) Potentially endemic to the Serra da Mocidade region; (PR) Punctual restricted localities; (TZ) Transition zones between Amazonia and other biomes. Altitudinal range: (L) Lowland (below 500 m asl); (U) Upland (above 500 m asl).

Taxon	Altitude (m asl)				Sampling method	Morphological measurements (mm)	Geographic distribution	Altitudinal range
	600	960	1,060	1,365				
**Amphibia**	89	28	101	14				
**Gymnophiona**			3					
**Rhinatrematidae**			1					
*Epicrionops* sp.			1		OE	SVL 83.5	PE ^§^	U^|^
**Siphonopidae**			2					
*Brasilotyphlus* sp.			2		OE		PE ^§,|^	U^|^
**Anura**	91	28	98	14				
**Allophrynidae**	1							
*Allophryne ruthveni* Gaige, 1926	1				AS	SVL 28.1	WD ^†^	L^‡‡‡^
**Aromobatidae**	25	4	23	8				
*Anomaloglossus apiau* Fouquet, Souza, Nunes, Kok, Curcio, Carvalho, Grant & Rodrigues, 2015	25	4	23	8	AS, OE	SVL 15–23	GH ^†,¶^	U^§,¶^
**Bufonidae**	3		3					
*Rhaebo guttatus* (Schneider, 1799)	3		3		AS	SVL 147–167	WD ^†^	L, U^‡‡‡^
*Rhinella marina* (Linnaeus, 1758)	5					SVL 21–44	WD ^†^	L^‡‡‡^
*Rhinella martyi* Fouquet, Gaucher, Blanc & Vélez-Rodriguez, 2007	1		13	1	AS, PT, OE	SVL 50–70	GS ^†,#^	L, U^#^
**Craugastoridae**	10	8	11	2				
Pristimantis aff. vilarsi	10	8	11	2	AS	SVL 17–56.9	GH ^†,§^	L, U^‡‡‡^
**Centrolenidae**	5	4	8					
Hyalinobatrachium aff. taylori	4	2	1		AS	SVL 18–21	PE ^†,§^	U^‡‡‡^
*Vitreorana ritae* (Lutz, 1952)	1	2	7		AS	SVL 17–22	GS, PR^†^	L, U^‡‡‡^
**Hemiphractidae**		2						
*Stefania* sp.		2			AS, OE	SVL 52, 54	PE ^†,§^	U^†^
**Hylidae**	11	10	40	3				
*Boana boans* (Linnaeus, 1758)	8	2	9		AS, OE	SVL 82–111	WD ^†^	L, U^‡‡‡^
*Boana multifasciata* (Günther, 1859)	1		2		AS	SVL 55–72	EA, TZ^†^	L, U^‡‡‡^
*Boana xerophylla* (Duméril & Bibron, 1841)			1		AS	SVL 57	GS ^†^	L, U^‡‡‡^
*Dendropsophus minutus* (Peters, 1872)		3	14		AS	SVL 20.5–27	WD ^†^	L, U^‡‡‡^
*Dendropsophus parviceps* (Boulenger, 1882)		1	5		AS	SVL 20–27	WD ^†^	L, U^‡‡‡^
*Osteocephalus taurinus* Steindachner, 1862	2	4	9	3	AS	SVL 64.5–90	WD, TZ^†^	L, U^‡‡‡^
**Leptodactylidae**	19							
*Adenomera andreae* (Müller, 1923)	1				AS	SVL 24	WD ^†,††^	L^‡‡‡^
*Leptodactylus guianensis* Heyer & de Sá, 2011	2				AS	SVL 103.7	GS ^†,††^	L^††,‡‡‡^
*Leptodactylus mystaceus* (Spix, 1824)	6				AS, OE	SVL 50–59	WD ^†,††^	L^††,‡‡‡^
*Leptodactylus petersii* (Steindachner, 1864)	1				AS	SVL 32.5	WD ^†,††^	L^††,‡‡‡^
*Physalaemus ephippifer* (Steindachner, 1864)	7				AS, PT	SVL 20–28	GS ^†,††^	L^††,‡‡‡^
**Ranidae**	11							
*Lithobates palmipes* (Spix, 1824)	11				AS	SVL 72–96	WD ^†^	L^‡‡‡^
**Reptilia**	58	2	13					
**Squamata**	43	2	13					
‘**Sauria**’	34	1	5					
**Gymnophthalmidae**	3							
*Cercosaura ocellata* Wagler, 1830	1				PT	SVL 56.5	EA ^‡,††††^	L^‡‡^
*Tretioscincus oriximinensis* Ávila-Pires, 1995	2				AS, GT	SVL 53, 55	GS, PR^‡,‡‡, ††††^	L, U^‡‡^
**Dactyloidae**	12		2					
*Anolis punctatus* Daudin, 1802			1		AS	SVL 83	WD ^‡,§§^	L, U^‡‡^
*Anolis fuscoauratus* d’Orbigny, 1837	11		1		AS	SVL 40–49	WD ^‡,§§^	L, U^‡‡^
*Anolis planiceps* Troschel, 1848	1				AS	SVL 40	GS ^‡,§§^	L, U^‡‡^
**Phyllodactylidae**	4							
*Thecadactylus rapicauda* (Houttuyn, 1782)	4				AS, GT, OE	SVL 92–115	WD ^‡,||^	L^‡‡^
**Sphaerodactylidae**	5							
*Pseudogonatodes guianensis* Parker, 1935	5				AS, GT	SVL 15–17	WD ^‡,||^	L^‡‡^
**Polychrotidae**	1							
*Polychrus marmoratus* (Linnaeus, 1758)	1				SG	SVL 133	WD ^‡,§§^	L^‡‡^
**Tropiduridae**	2	1	3					
*Plica plica* (Linnaeus, 1758)	2	1	3		AS, PT, SG, OE	SVL 52–141	WD ^‡,§§^	L, U^‡‡^
**Teiidae**	3							
*Ameiva ameiva ameiva* (Linnaeus, 1758)	3				SG	SVL 52–85	WD ^‡,‡‡^	L^‡‡^
**Scincidae**	4							
*Mabuya nigropunctata* (Spix, 1825)	4				PT, SG	SVL 72–105	WD ^‡,‡‡^	L^‡‡^
**Serpentes**	9	1	8					
**Colubridae**	2		2					
*Atractus riveroi* Roze, 1961			3		PT	SVL 229–290, CL 29.1–54.5	GH ^‡,¶¶^	U^¶¶^
*Chironius fuscus* (Linnaeus, 1758)	6		4		AS	SVL 565, 603, CL 320, 357	WD ^‡,##^	L, U^§§§^
*Chironius septentrionalis* Dixon, Wiest & Cei, 1993			1		AS	SVL 1,480, CL 350	GH ^‡,##^	L, U^‡‡‡,§§§^
*Drymobius rhombifer* (Günther, 1860)	1				AS	SVL 365, CL 131	WD ^‡,##^	L, U^‡‡‡^
*Dipsas catesbyi* (Sentzen, 1796)	1				OE	SVL 229, CL 89	WD ^‡,†††^	L, U^‡‡‡^
*Dipsas indica indica* Laurenti, 1768	1				AS	SVL 520, CL 209	WD ^‡,##^	L, U^‡^
*Dipsas pavonina* Schlegel, 1837	1				OE	SVL 275, CL 95	EA, AN^‡,†††^	L, U^‡‡‡^
*Imantodes cenchoa* (Linnaeus, 1758)	1				AS	SVL 713, CL 315	WD ^‡,##^	L^‡^
*Xenodon rabdocephalus rabdocephalus* (Wied, 1824)	1				AS	SVL 440, CL 399	WD ^‡,##^	L^##^
**Elapidae**			1					
*Micrurus remotus* Roze, 1987			1		OE	SVL 42.7, CL 7	WA ^‡,##^	L, U^##^
**Viperidae**	2	1	2					
*Bothrops atrox* (Linnaeus, 1758)	1		1		AS, PT	SVL 93.5, 952, CL 17.5, 170	WD ^‡,##^	L, U^##^
*Bothrops bilineatus bilineatus* (Wied, 1821)		1	1		AS	SVL 450, 626, CL 80, 104	WD ^‡,##^	L, U^##^
*Lachesis muta muta* (Linnaeus, 1766)	1				AS	SVL 1,575, CL 175	WD ^‡,##^	L^|||^
**Testudines**	13							
**Chelidae**	11							
*Mesoclemmys gibba* (Schweigger, 1812)	10				TN, HM	CAL 109–189, CW 84–138	WD ^‡,¶¶¶^	L^¶¶¶^
*Platemys platycephala melanonota* Ernst, 1984	1				OE	-	WD ^‡,¶¶¶^	L^¶¶¶^
**Testudinidae**	2							
*Chelonoidis denticulatus* (Linnaeus, 1766)	2				OE	-	WD ^‡,¶¶¶^	L^¶¶¶^
**Crocodylia**	2							
**Alligatoridae**	2							
*Paleosuchus trigonatus* (Schneider, 1801)	2				AS	SVL 160, 675	WD ^‡,###^	L, U^###^

^†^ Frost 2017, ^‡^ Uetz and Hošek 2017, ^§^ Authors personal observations, ^|^ Maciel and Hoogmoed 2011, ^¶^ Fouquet et al. 2015, ^#^ Fouquet et al. 2007b, ^††^ de Sá et al. 2014, ^‡‡^ Ávila-Pires 1995, ^§§^ Ribeiro-Júnior 2015a, ^||^ Ribeiro-Júnior 2015b, ^¶¶^ Passos et al. 2013, ^##^ Wallach et al. 2014, ^†††^ Lima and Prudente, 2009, ^‡‡‡^ IUCN 2016, ^§§§^ Dixon et al. 1993, ^|||^ Vial and Jimenez-Porras 1967, ^¶¶¶^ van Dijk et al. 2014, ^###^ Magnusson and Campos 2010, ^††††^ Ribeiro-Júnior and Amaral 2017.

**Figure 10.**
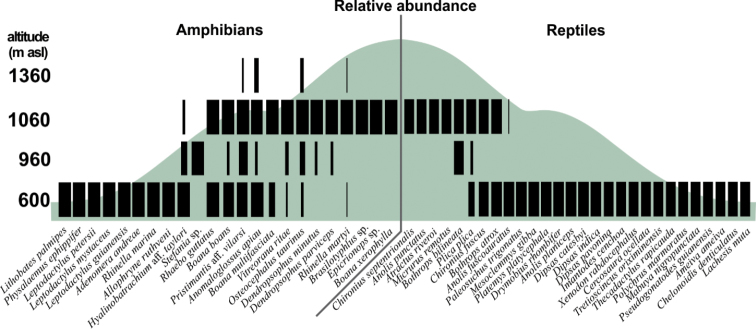
Altitudinal species variation. Altitudinal variation in species composition and relative abundance (width of the black bars) of amphibians and reptiles from our sampling at the Serra da Mocidade mountain range. Note that some species were exclusively recorded in a given altitudinal level while other are altitudinal generalists.

The number of specimens recorded during sampling was similar in both camps (149 at base camp #1 vs. 158 at base camp #2), with some species exclusively recorded at distinct altitudinal levels, as with frogs of the genus *Leptodactylus* Fitzinger, 1826 only recorded at 600 m asl or the snakes *Micrurus remotus* Roze, 1987 and *Chironius septentrionalis* Dixon, Wiest & Cei, 1993 only recorded at 1,060 m asl (Fig. 10). Regarding sampling methods, the colubrid *Atractus riveroi* Roze, 1961 and the gymnophthalmid *Cercosaura ocellata* Wagler, 1830 were exclusively recorded using pitfall traps, a method that recorded a total of 15 specimens. Active surveys recorded 252 specimens, eight specimens were collected using shotguns, three using glue traps, ten using trammel nets and hook with bait, and 17 by occasional encounters, including all three caecilians.

Advertisement calls were obtained for a few anuran species, and they were useful to assign or confirm species identification, such as *Dendropsophus minutus* (Peters, 1872), *Rhinella martyi* Fouquet, Gaucher, Blanc & Vélez-Rodriguez, 2007, *Boana boans* (Linnaeus, 1758) and *Anomaloglossus apiau* Fouquet, Souza, Nunes, Kok, Curcio, Carvalho, Grant & Rodrigues, 2015. We obtained DNA sequences from 74 specimens of taxonomically confusing taxa (10 species) to conduct our phylogenetic analyses. These analyses revealed that some species initially thought by us to be new taxa represent taxa already described, for instance *A. apiau*, *R. martyi*, *Dendropsophus parviceps* (Boulenger, 1882), *Vitreorana ritae* (Lutz, 1952) and *Tretioscincus oriximinensis* Ávila-Pires, 1995. Additionally, molecular data indicated the existence of some new candidate species (Padial et al. 2010), such as *Stefania* sp. *Epicrionops* sp. and *Brasilotyphlus* sp. and other taxa that require further studies to clarify their taxonomic status, such as Pristimantis aff. vilarsi and Hyalinobatrachium aff. taylori.

### Diversity and biogeographical affinities

The species richness of Serra da Mocidade is comparable to that recorded for other mountain ranges in the Guiana Shield highlands region, which harbour a low number of species compared to Guiana Shield lowland forest habitats (Table 2). Regarding the species composition, the amphibians and reptile assemblages of the Serra da Mocidade were evidenced in a intermediate position between the Guiana Shield highlands and lowland forest assemblages, which are distinct from the cluster generated by the lowland open habitat assemblages (*R*² = 0.74, stress = 0.14). We note a high divergence in species composition of the mountain ranges included in the analyses, because many of these mountains harbour several endemic species. Among these mountain ranges, the herpetofauna of Serra da Mocidade is more similar to that occurring at the geographically close Apiaú and Neblina, as well as to that occurs at the uplands of Nassau and Lely plateaus, which reach lower altitudes than the others mountain ranges included in this analyses (Fig. 9c).

**Table 2.** Species richness. Herpetofaunal richness variation at some mountain ranges in the Guiana Shield highlands (including data for all altitudinal levels) and at lowland habitats in the region (compiled results of inventories). Data are presented as raw species number/percentage of total herpetofauna and references are detailed in the text.

Mountain range	Amphibians	Reptiles	Total
Mocidade	23/0.45	28/0.55	51
Apiaú	23/0.48	25/0.52	48
Duida	10/0.38	16/0.62	26
Marahuaka	14/0.74	5/0.26	19
Auyantepui	14/0.35	26/0.65	40
Chimantá	8/0.42	11/0.58	19
Guaiquinima	11/0.33	22/0.67	33
Neblina	51/0.45	62/0.55	113
Los Testigos	4/0.67	2/0.33	6
Lema	31/0.53	28/0.47	59
Guanay	4/0.57	3/0.43	7
Yaví	3/0.50	3/0.50	6
Tamacuari	7/0.64	4/0.36	11
Yutajé-Corocoro	6/0.60	4/0.40	10
Roraima	15/0.65	8/0.35	23
Sarisariñama	16/0.44	20/0.56	36
Nassau, Lely	32/0.59	22/0.41	54
**Lowland habitats**			
Forests	72/0.42	100/0.58	172
Open habitats	40/0.54	33/0.46	73

This same mixed influence is corroborated regarding biogeographical regions, as the composition of the amphibian assemblage was most strongly influenced by widely distributed Amazonian forest lineages (47%), but also included species restricted to, or typical for, the Guiana Shield (22%), Guiana Shield highlands (8%) and Eastern Amazonia (4%). The reptile assemblage composition showed a similar pattern, with most species widely distributed in Amazonia (75%) and smaller proportions of exclusive lineages from the Guiana Shield (7%), Guiana Shield highlands (7%), Eastern Amazonia (7%) and Western Amazonia (3.5%). Some of the taxa we collected are potentially endemic to the Serra da Mocidade mountain range, such as *Stefania* sp., *Epicrionops* sp., *Brasilotyphlus* sp. and Hyalinobatrachium aff. taylori (Table 1).

Most of the amphibian species we recorded on Serra da Mocidade occur across wide altitudinal ranges along their geographical distribution (43%). Several species are typical from the Amazonian lowlands (35%), and some are typical of the uplands (22%). The reptilian assemblage showed a similar altitudinal pattern: most of the Serra da Mocidade species are generalists in terms of altitudinal range (53.5%) or lowland forms (43%), and few are typical of the uplands (3.5%). When we analysed the distinct assemblages recorded at 600 m asl and above 900 m asl, we found different distribution patterns at a finer scale: at 600 m asl, assemblages were mainly composed of lowland species (47% of amphibians and 52% of reptiles) and altitudinal generalists (42% of amphibians and 48% of reptiles). Above 900 m asl, no elements from lowland forests were recorded for either taxonomic group and assemblages were composed of upland species (33% of amphibians and 11% of reptiles) and altitudinal generalists (67% of amphibians and 89% of reptiles) (Fig. 10).

### Remarkable records

Accounts of the remarkable species are provided below, with comments on ecology, taxonomy, morphology, evolutionary distinctiveness, and biogeography.

**Figure 11.**
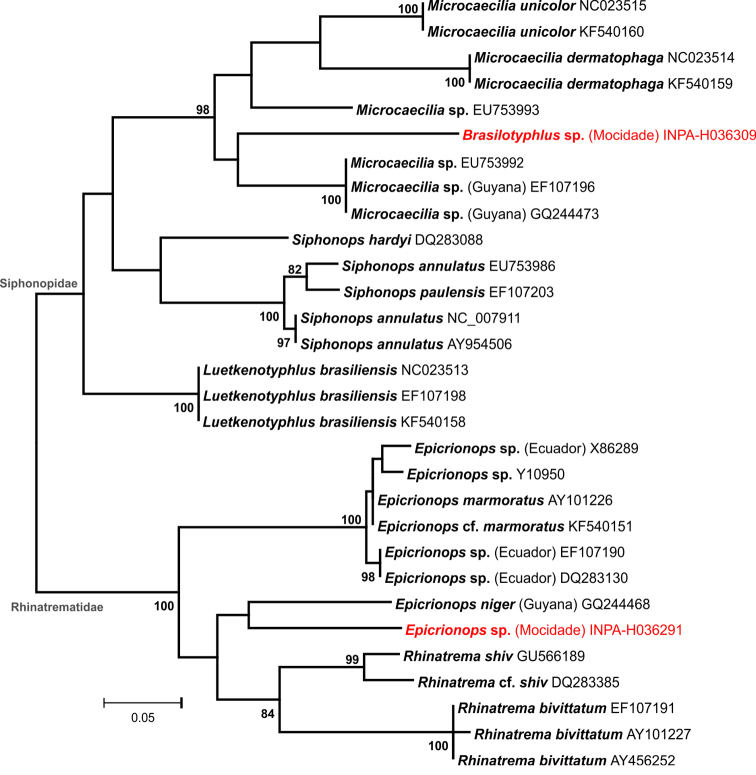
Phylogenetic relationships of caecilians. Maximum likelihood phylogenetic tree of caecilians families Siphonopidae and Rhinatrematidae, based on a 468bp fragment of the 16S mtDNA. Only bootstrap values >80% are shown (5,000 replicates). The GenBank accession numbers appear after the names of downloaded sequences, and specimens from the Serra da Mocidade are highlighted.

*Epicrionops* sp. – The genus *Epicrionops* Boulenger, 1883 is distributed in mountainous habitats of the Andes (seven species) and the Guiana Shield highlands [*Epicrionops niger* (Dunn, 1942)] (Frost 2017). However, the generic position of *E. niger* is uncertain, as recent studies have shown this species to be genetically closer to species in the genus *Rhinatrema* (distributed mainly throughout the Guiana Shield) than to Andean species of *Epicrionops* (Pyron and Wiens 2011, San Mauro et al. 2014). The juvenile specimen recorded at Serra da Mocidade (Fig. 4h) is the first of the genus recorded from Brazil and has a high genetic distance to *E. niger* from Guyana (>15% on the 16S fragment used, Fig. 11). Considering such high molecular divergence and degree of geographical isolation, this taxon represents a new candidate species, needing further studies and samples for its formal description.

*Brasilotyphlus* sp. – The genera *Microcaecilia* Taylor, 1968 and *Brasilotyphlus* Taylor, 1968 (possibly synonymous by lack of diagnosability–see Maciel and Hoogmoed 2011) are distributed throughout eastern Amazonia, with highest diversity in the Guiana Shield. We found two caeciliid specimens at Serra da Mocidade, both at 1,060 m asl, possessing a diastema between palatine and vomerine teeths, the only known morphological characteristic diagnostic for the genus *Brasilotyphlus* (Maciel et al. 2009, Wilkinson et al. 2013). However, some morphological characteristic distinguish those specimens for other *Brasilotyphlus*, e.g. the lower number of primary annulus (less than 140 vs. more than 141 in the two known species). Although no *Brasilotyphlus* or *Microcaecilia* is known from the Brazilian side of the Guiana Shield highlands region, there is evidence that at least two undescribed species occur there, and one of them is in description process (Maciel and Hoogmoed 2011, Pedro Nunes pers. comm.). Our molecular analyses showed this taxon to be highly divergent from other *Microcaecilia* with available sequences in GenBank, with a minimum genetic distance of 17% on the 16S fragment used (Fig. 11). Based on these morphological and molecular results, we consider the *Brasilotyphlus* collected at Serra da Mocidade as a new candidate species.

**Figure 12.**
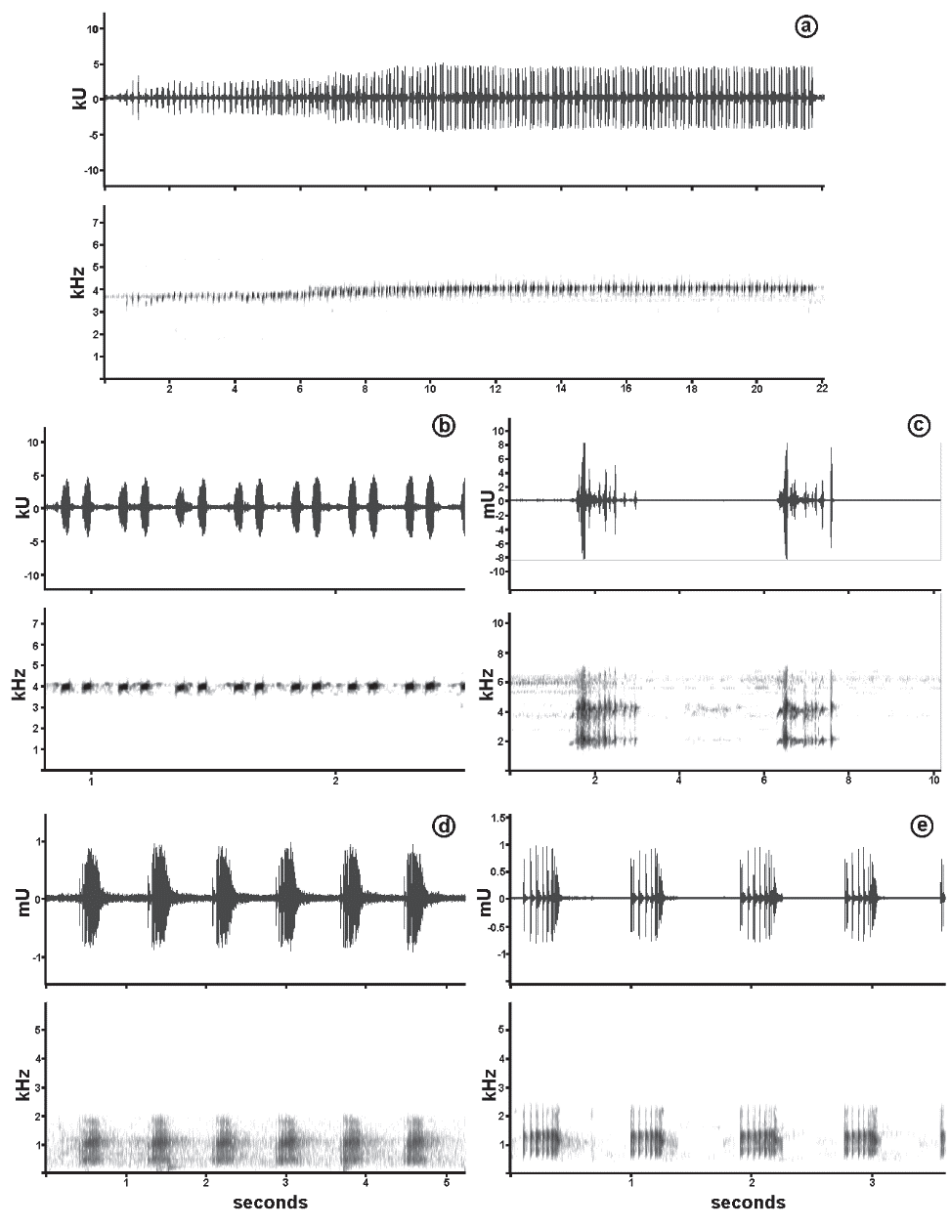
Bioacoustical data. Oscillograms (upper graphs) and sonograms (lower graphs) of advertisement calls of species recorded in the Serra da Mocidade mountain range. **a** *Anomaloglossus apiau*, zoomed at **b** showing the paired pulses **c** *Dendropsophus minutus* **d** *Boana boans* **e** *Rhinella martyi*.

**Figure 13.**
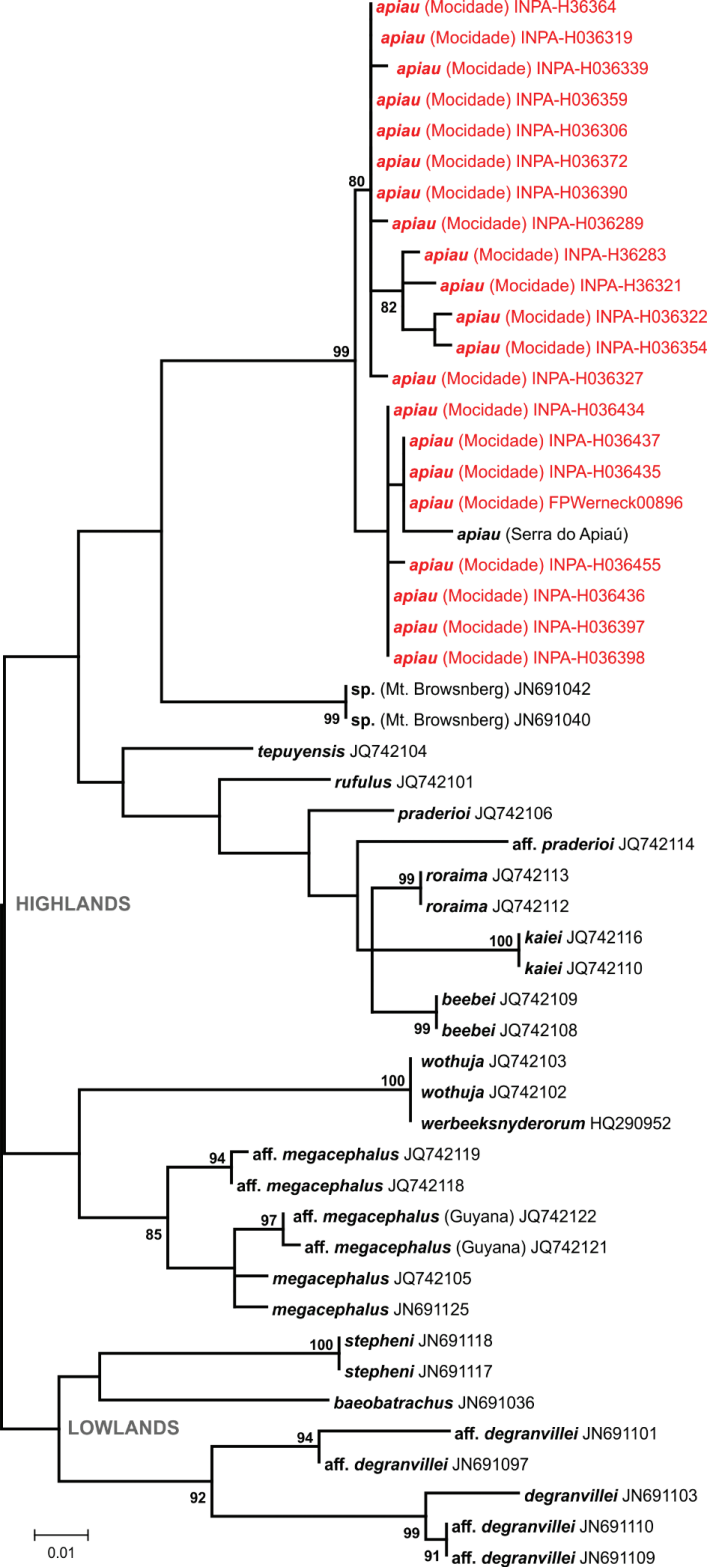
Phylogenetic relationships of *Anomaloglossus*. Maximum likelihood phylogenetic tree of *Anomaloglossus* species, based on a 361bp fragment of the 16S mtDNA. Only bootstrap values >80% are shown (5,000 replicates). The GenBank accession numbers appear after the names of downloaded sequences, and specimens from the Serra da Mocidade are highlighted.

*Anomaloglossus apiau* – The high endemism levels of the small cryptically coloured terrestrial frogs of the genus *Anomaloglossus* recorded from Guiana Shield highlands mountains (Kok et al. 2012, Vacher et al. 2017), together with the geographical isolation of Serra da Mocidade, led us to the immediately suspect that the population found in this mountain range was a new taxon. However, detailed analyses showed that their morphological (Fig. 3a,b), acoustic (Fig. 12, Table 3) and molecular variation (Fig. 13) are consistent with the description of *A. apiau* (Fouquet et al. 2015a), a species previously known only from its type locality, at Serra do Apiaú, a mountain range distant ca. 80 km northeast of Serra da Mocidade. Here we extend the known geographical range of this species. We found the species in all altitudinal levels sampled at Serra da Mocidade, near rocky streams with rapids and waterfalls (Fig. 8d, e).

**Table 3.** Quantitative bioacoustical results. Summary of bioacoustical analyses of advertisement calls of some species recorded from the Serra da Mocidade mountain range, with literature data for comparison. Data are presented as mean, with associated amplitude (–) or standard deviation (±).

Species	Locality (References)	Call structure	Call duration (s)	Inter-call interval (s)	Pulse duration (s)	Pulse rate (pulses/s)	Dominant frequency (kHz)
*Anomaloglossus apiau*	Serra da Mocidade, RR, Brazil	Long trill	19.07 (±4.89)	15.76 (±4.12)	0.039 (±0.002)	8.4 (±0.67)	4.109 (±0.148)
*Anomaloglossus apiau*	Serra do Apiaú, RR, Brazil^†^	Long trill	19.56 (±11.05)	11.17 (±7.24)	0.033 (±0.005)	11.27 (±0.55)	4.334 (±0.129)
*Dendropsophus minutus*	Serra da Mocidade, RR, Brazil	Single note	0.09 (0.04–0.18)	13 (11.2–17.4)	-	-	3.72 (2.1–4.5)
*Dendropsophus minutus*	Different localities^‡^	Single note	0.11 (0.03–0.2)	14.7 (11.1–18.3)	-	-	3.75 (2.2–5.3)
*Boana boans*	Serra da Mocidade, RR, Brazil	Long train	0.34 (0.18–0.51)	1.51 (1.27–2.10)	-	-	1.036 (0.9–1.1)
*Boana boans*	Different localities^|^	Long train	0.42 (0.18–1.19)	2.05 (0.57–4.7)	-	-	0.648 (0.2–1.1)
*Rhinella martyi*	Serra da Mocidade, RR, Brazil	Series of pulses	0.302 (±0.026)	0.61 (±0.18)	0.015 (±0.004)	-	1.237 (±0.03)
*Rhinella martyi*	Guiana Shield lowlands^§^	Series of pulses	0.295 (±0.013)	-	0.009 (±0.001)	-	1.169 (±0.04)

^†^ Fouquet et al. 2015, ^‡^ Cardoso and Haddad 1984, Donnelly and Myers 1991, Morais et al. 2012, ^|^ Hoogmoed 1990, ^§^ Fouquet et al. 2007b.

The population of Serra da Mocidade had adult males with 15–20 mm SVL, and females slightly larger than originally described for *A. apiau* (19–23 mm SVL) (see Fouquet et al. 2015a). As in the population from Serra do Apiaú, specimens from Serra da Mocidade had a high intraspecific polymorphism in colour pattern, and strong sexual dichromatism (Fig. 3a, b). The advertisement call has the same temporal and spectral structure as reported in the species description (long series of paired notes, followed by intervals of silence) (Fig. 12, Table 3). Additionally, the population from Serra da Mocidade occurs within the altitudinal range cited in the original description for the type locality (500–1,400 m asl). Molecular analyses confirmed the similarity between the two populations, with the sample of *A. apiau* from the type locality nested within the Serra da Mocidade clade (Fig. 13). Two subclades from Serra da Mocidade are separated by a low genetic distance (maximum 2% on the 16S fragment used) and occur at different altitudes and drainages. However, this difference is most likely due to natural intraspecific variation, since the genetic distance between populations of *A. apiau* from the type locality and Serra da Mocidade is also below 2%.

This record of *A. apiau* from the Serra da Mocidade (first outside the type locality) is remarkable, since the species was not recorded from the mountain range of the Serra da Maroquinha, located at less than 5 km southeast from the Serra do Apiaú. New studies on the cryptic diversity, evolution and biogeography of the genus *Anomaloglossus* (such as Vacher et al. 2017) should reveal the species limits, geographical patterns and drivers of the diversification in low- and uplands of the Guiana Shield.

*Rhinella martyi* – *Rhinella martyi* has a confusing taxonomic history, as do many of the small-medium terrestrial forest bufonids in the *Rhinella margaritifera* group (dos Santos et al. 2015). Until recently, this taxon was allocated in the problematic taxon *Rhinella margaritifera* (Laurenti, 1768), but Fouquet et al. (2007a) recognized the presence of distinct lineages occurring in the Guiana Shield. Two of these lineages were later described as the new species *R. lescurei* and *R. martyi* (Fouquet et al. 2007b), but the specific status of the latter was rejected by Ávila-Pires et al. (2010). However, awaiting further integrative revisions of the group, the name *R. martyi* was retained (Frost 2017). Both the neotype designated to *Rhinella margaritifera* (Lavilla et al. 2013) and the holotype recently rediscovered (Lavilla et al. 2017), have a different morphology from populations named as *R. martyi* (e.g., by having larger cranial crests and body size). Apparently, *R. martyi* is widely distributed along the northern Guiana Shield, occurring along a broad altitudinal range (Fouquet et al. 2007b).

Individuals of *R. martyi* were found on leaf-litter inside primary forests, and calling males on the banks of streams, mostly at 1,060 m asl (Fig. 3d). Morphological variation among specimens from the Serra da Mocidade mostly fits with the variation proposed in the original species’ description, except in a lower variation in adult body size (at Serra da Mocidade, males 50–60 mm SVL, females 62–70 mm SVL), smaller height of supratympanic crests and the bony knob at angle of jaws less developed. The advertisement call recorded at Serra da Mocidade also differs slightly from the species description, with longer pulses and higher dominant frequency (Fig. 12, Table 3). However, the overall morphological and acoustic variation in *R. martyi* is still being clarified (A. Fouquet pers. comm.). Elucidation of the taxonomic status of populations of *Rhinella margaritifera* group from the Guiana Shield still depends on broader integrative revisions.

Pristimantis aff. vilarsi – *Pristimantis* is one of the most speciose genus of vertebrates (Padial et al. 2014, Frost 2017). Such high diversity is accompanied by a problematic taxonomy and difficulties in species delimitation (Padial et al. 2014). In the Guiana Shield highlands region, several lineages of *Pristimantis* diversified into lowlands and highland forms (Kok et al. 2011). We found a species from the *Pristimantis conspicillatus* group at the Serra da Mocidade (Fig. 4g). It may represent a new taxon, but its taxonomic status is being evaluated in a broader sense in relation to other *Pristimantis*. Our molecular analyses showed that samples from the Serra da Mocidade are more similar to *Pristimantis vilarsi* (Melin, 1941, redescribed in Barrio-Amorós and Molina 2006) and *Pristimantis zeuctotylus* (Lynch & Hoogmoed, 1977), both species of the *P. conspicillatus* group from the lowlands and uplands of Guiana Shield. The population from Serra da Mocidade has morphological details that distinguish it from these two genetically-related species, as for example the adult body size (SVL): 22–33 mm in males and 31–49 mm in females of *P. vilarsi*, 20–29 mm in males and 30–43 mm in females of *P. zeuctotylus* (Barrio-Amorós and Molina 2006) and 36–46 mm in males and 48–57 mm in females from Serra da Mocidade. Pristimantis aff. vilarsi occurs across the altitudinal range sampled at Serra da Mocidade and was one of the most abundant anurans in our sampling (along with *A. apiau*). It was recorded on leaf-litter of primary dense forests, but also inhabits rocky outcrops within streams with fast flowing water (Fig. 8d, e).

**Figure 14.**
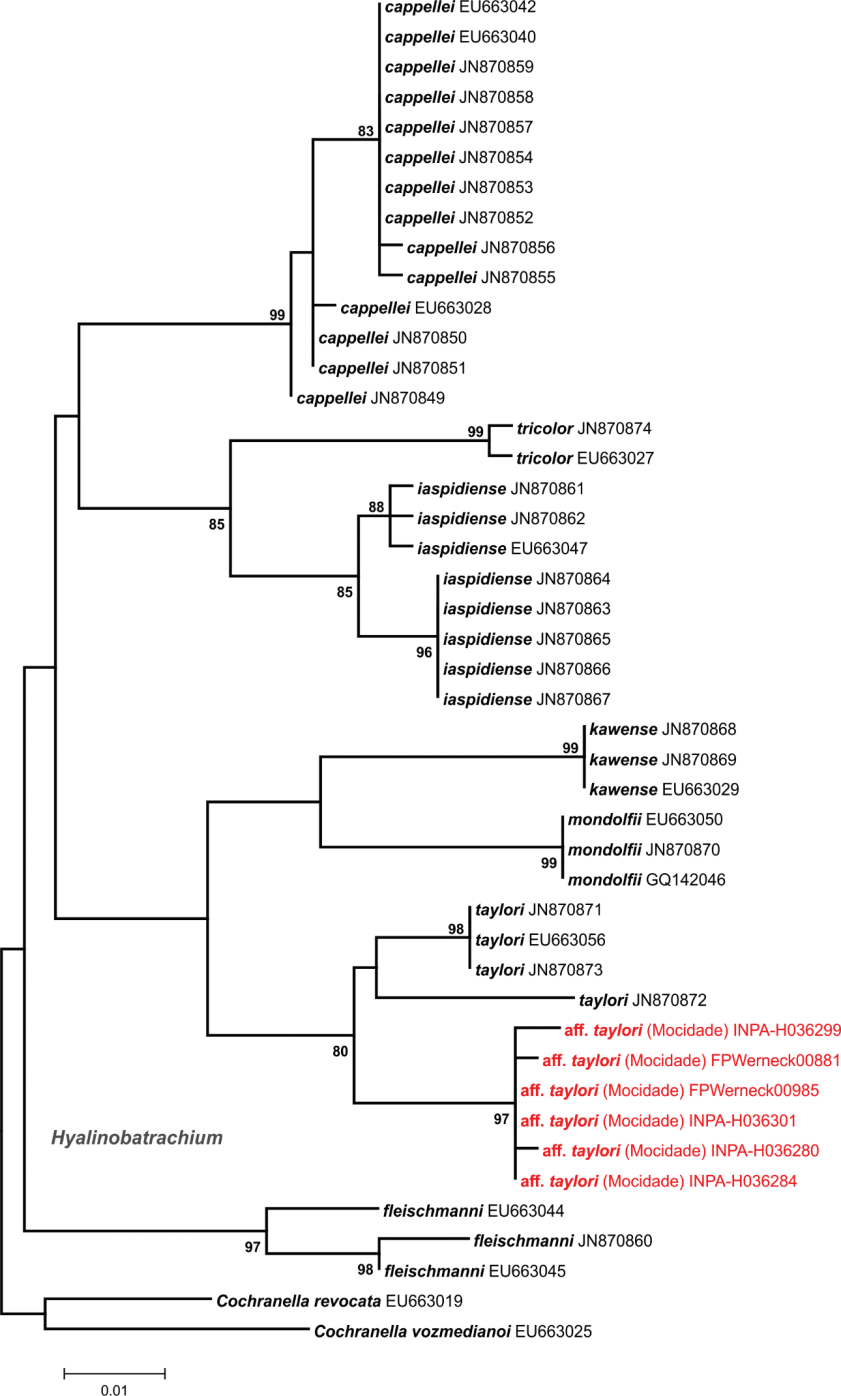
Phylogenetic relationships of *Hyalinobatrachium*. Maximum likelihood phylogenetic tree of *Hyalinobatrachium* species from Guiana Shield, based on a 465bp fragment of the 16S mtDNA. Only bootstrap values >80% are shown (5,000 replicates). The GenBank accession numbers appear after the names of downloaded sequences, and specimens from the Serra da Mocidade are highlighted.

Hyalinobatrachium aff. taylori – The species of *Hyalinobatrachium* from Serra da Mocidade (Fig. 3e) was mainly found at 600 m asl. Most of specimens collected were juveniles, with few adults varying in body size from 18 to 21 mm SVL. The species is morphologically similar to *Hyalinobatrachium taylori* (Goin, 1968), a taxon widely distributed within the northern Guiana Shield (Castroviejo-Fisher et al. 2011). Both have similar body sizes, snout shape (round in dorsal view and sloping in lateral view), life colouration of dorsum (dark green with small white spots), eyes (grey, black reticulated), hands and feet (yellowish-orange) (Castroviejo-Fisher et al. 2011). However, specimens from Serra da Mocidade differ from *H. taylori* by having white bones instead of green (Señaris and Ayarzagüena 2005, Guayasamin et al. 2009, Castroviejo-Fisher et al. 2011). It is possible that this difference represents an intraspecific variation, since the genetic distance on the 16S fragment used was less than 4% between both taxa (Fig. 14), but bone colouration is a strong character in the genus taxonomy, and green bones is a rare character in *Hyalinobatrachium* (two species), but common in other centrolenid genera, such as *Vitreorana* and *Centrolene* (Guayasamin et al. 2009). In addition, Serra da Mocidade is located far from the known geographical range of *H. taylori*, and the occurrence of this species at this locality would be the first record of the species in Brazil.

The diversity of *Hyalinobatrachium* in the Guiana Shield highlands region appears underestimated, since some lineages were recently described as new species after an integrative revision (Castroviejo-Fisher et al. 2011), and the evolution of lineages in the *H. taylori* complex seem strongly influenced and structured by the altitudinal levels in which they occur, with clades from lowlands and highlands separated by low genetic distances (Castroviejo-Fisher et al. 2011). Considering the uncertainties, and waiting for the results of future detailed studies, we opted to keep this taxon as H. aff. taylori.

**Figure 15.**
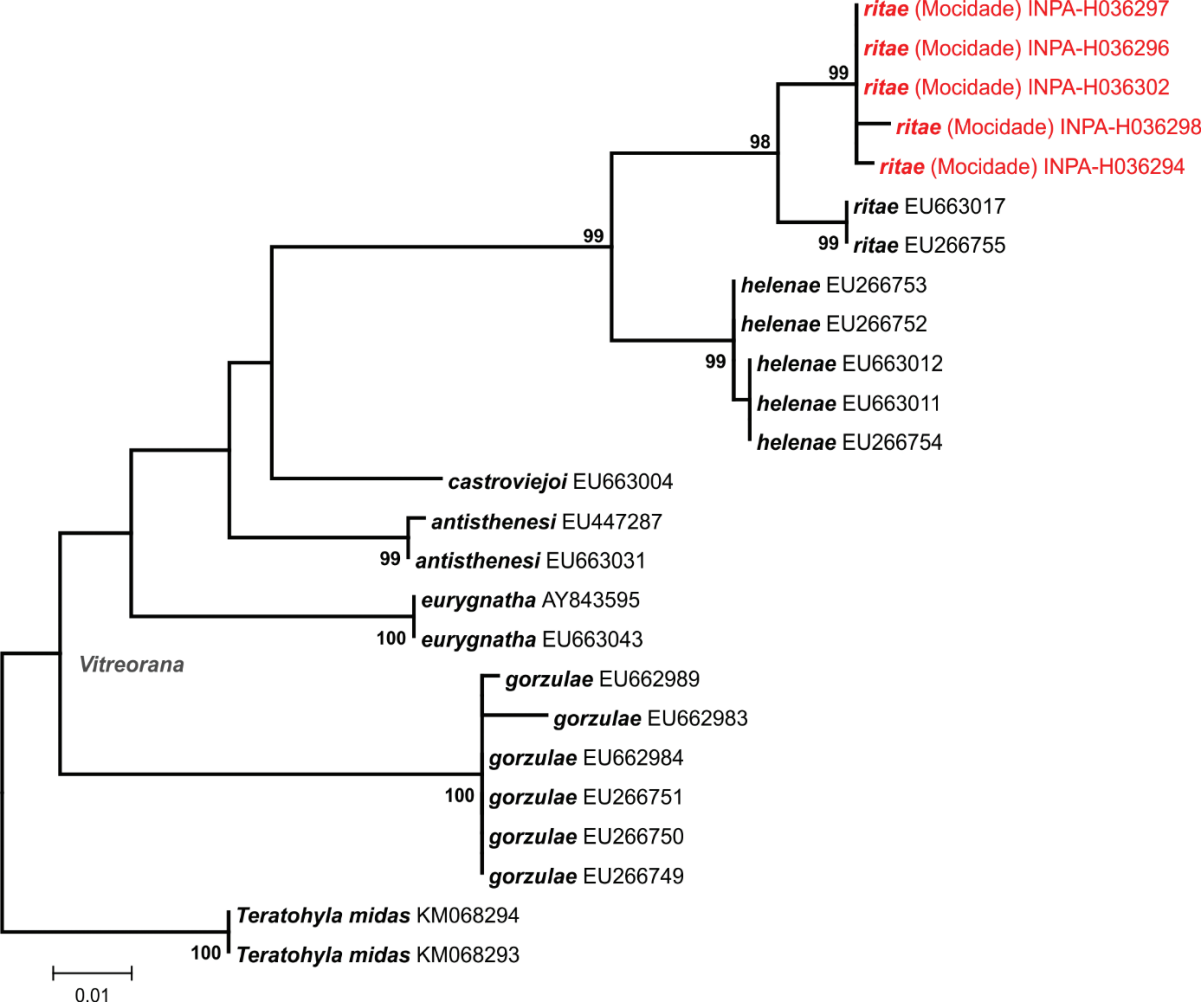
Phylogenetic relationships of *Vitreorana*. Maximum likelihood phylogenetic tree of *Vitreorana* species, based on a 496bp fragment of the 16S mtDNA. Only bootstrap values >80% are shown (5,000 replicates). The GenBank accession numbers appear after the names of downloaded sequences, and specimens from the Serra da Mocidade are highlighted.

*Vitreorana ritae* – The small glassfrog *Vitreorana ritae* is apparently widely distributed in the Guiana Shield (Guayasamin et al. 2009), occupying a wide altitudinal range (see Cisneros-Heredia 2013 for taxonomical accounts and synonymization of *V. oyampiensis* with *V. ritae*). However, single-site records in western Amazonia and south of the Amazon River cloud the delineation of the total species’ range and possible connections between populations. The species was found at Serra da Mocidade (Fig. 3f) in riparian vegetation at three altitudinal levels, but with greatest abundance at 1,060 m asl. Specimens from Serra da Mocidade are differentiated from *Vitreorana helenae* (Ayarzagüena, 1992), a morphologically similar taxon from the Guiana Shield highlands region that has yellow eyes and a lime-green dorsum (eyes predominantly gray and a darker green dorsum in *V. ritae*) (Guayasamin et al. 2008). Molecular data also grouped sequences from the Serra da Mocidade with sequences of *V. ritae* from other Guiana Shield localities with less than 2% of genetic distance on the 16S fragment used, while the distinction between *V. ritae* and *V. helenae* lies between 4 and 5% (Fig. 15).

**Figure 16.**
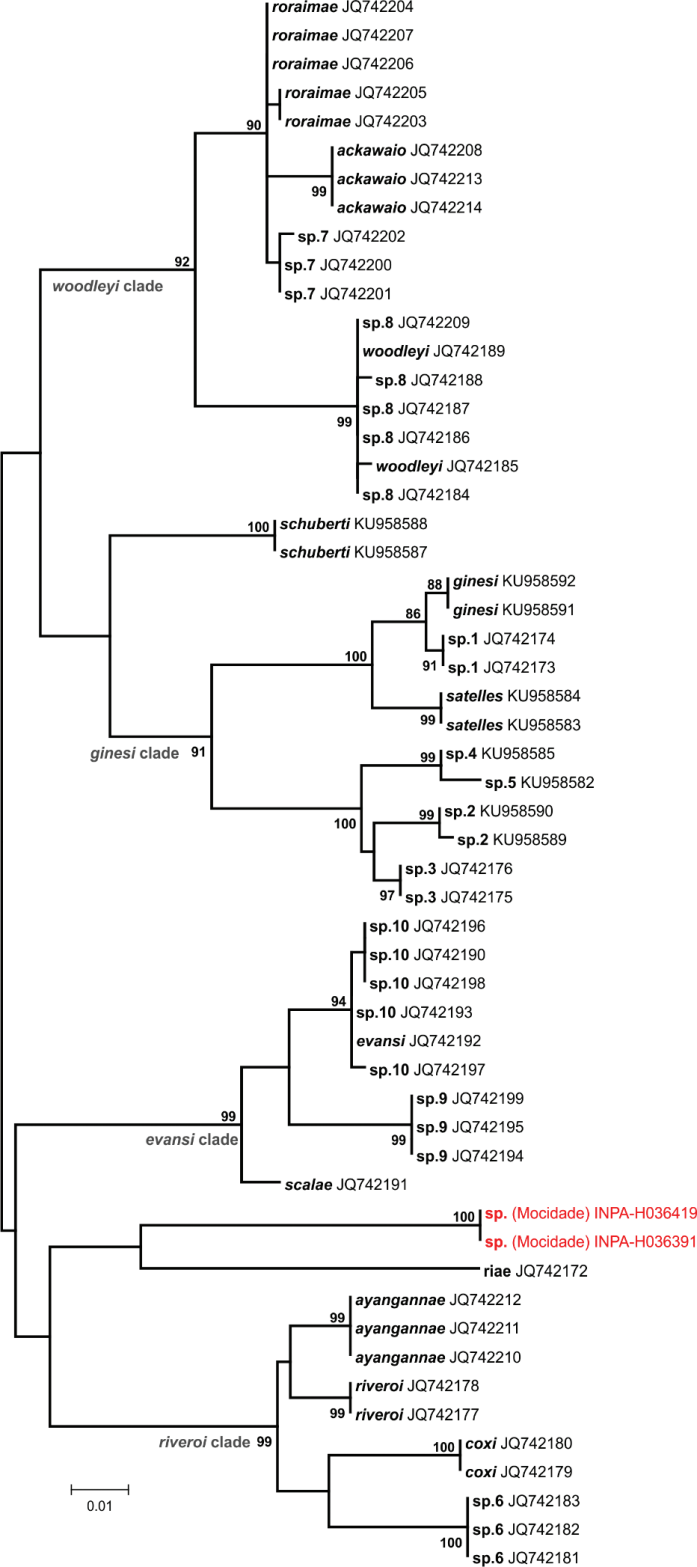
Phylogenetic relationships of *Stefania*. Maximum likelihood phylogenetic tree of *Stefania* species, based in a 536bp fragment of the 16S mtDNA. Only bootstrap values >80% are shown (5,000 replicates). The GenBank accession numbers appear after the names of downloaded sequences, species numbers of undescribed taxa follow Kok et al. (2017) and specimens from the Serra da Mocidade are highlighted.

*Stefania* sp. – The marsupial frog genus *Stefania* have an evolutionary history intrinsically linked to the evolution of the Guiana Shield highlands landscape, as the genus has a geographical range restricted to this region and high endemism levels in distinct mountain ranges (Duellman and Hoogmoed 1984, Salerno et al. 2012, Duellman 2015, Kok et al. 2016, 2017). We found two adult *Stefania* at 960 m asl on rocky outcrops in rapidly flowing streams (Fig. 8d, e). The specimens from Serra da Mocidade (Fig. 4c) have a large body size (52–54 mm SVL), head wider than long (head length / width = 0.93–0.94), snout rounded in dorsal and lateral views, shagreneed dorsum, basal toe webbing, colour pattern in life dorsally yellowish-brown with large rectangular blotches, ventrally gray (lighter in the abdomen) with large whitish patches, and large t-shaped finger and toe discs. The combination of these morphological characters distinguishes these specimens from other congeners (see variation in Rivero 1970, Myers and Donnelly 1997, MacCulloch and Lathrop 2002, Carvalho et al. 2010). The most similar species considering the external morphology is also the one with the closest known geographical distribution (*Stefania tamacuarina* Myers & Donnelly, 1997, which occurs ca. 300 km distant to the west of the Serra da Mocidade) (Caramaschi and Niemeyer 2005), however these taxa slightly differ in length and shape of snout, dorsal and ventral colouration pattern, as well as size of eye and hands (Myers and Donnelly 1997). The morphological divergence compared to the known congeners is sustained by a high genetic distance (>10% on the 16S gene fragment used), even considering other undescribed taxa detected in a recent phylogenetic analyses of the genus (Kok et al. 2017) (Fig. 16). The population of *Stefania* sp. from Serra da Mocidade is apparently isolated from known ranges of both described and undescribed species, and when confirmed as a new species, will be the third known *Stefania* endemic to a granitic mountain, while other taxa in this genus mainly occur at summits of sandstone mountains (Myers and Donnelly 1997). While new specimen samples (especially adults of *S. tamacuarina*) and DNA sequences are not available, we opted to keep this taxon as a candidate species.

*Dendropsophus minutus* – The species complex of small arboreal *Dendropsophus minutus* hylids is widely distributed in South America east of Andes, occupying a broad altitudinal range (Frost 2017). The evolutionary history of this complex has been recently revised, showing multiple distinct lineages throughout its wide distribution (Gehara et al. 2014). Several distinct cryptic lineages occur in the Guiana Shield (already noted by Hawkins et al. 2007), which suggests the elevation of the synonymized taxon *Dendropsophus goughi* [species described by Boulenger (1911) and synonymized to *D. minutus* by Lutz (1973)] to specific level for at least one of these lineages. While new studies do not clarify this taxonomic issue, the available name for the population recorded in the Serra da Mocidade is *D. minutus* (Fig. 3g). The specimens recorded share the presence of distinct light stripes in the cloacal region and in some specimens in the heels, and emitted mainly territorial calls (type B *sensu* Cardoso and Haddad 1984), with acoustic parameters within the known variation for the species (Fig. 12, Table 3).

**Figure 17.**
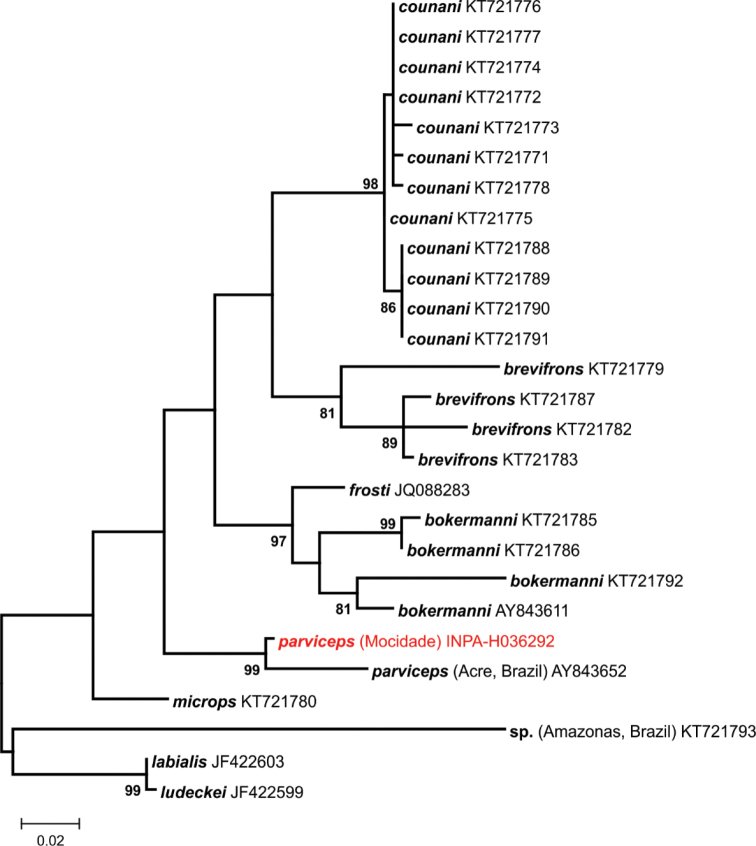
Phylogenetic relationships of *Dendropsophus*. Maximum likelihood phylogenetic tree of some species from *Dendropsophus parviceps* clade, based on a 350bp fragment of the 16S mtDNA. Only bootstrap values >80% are shown (5,000 replicates). The GenBank accession numbers appear after the names of downloaded sequences, and specimens from the Serra da Mocidade are highlighted.

*Dendropsophus parviceps* – Recent molecular studies on the evolution of the genus *Dendropsophus* found a polyphyletic *Dendropsophus parviceps* species group *sensu* Faivovich et al. 2005 (Fouquet et al. 2011, 2015b, Motta et al. 2012), which harbours small cryptically coloured treefrogs. Recently, a new species of this group (*Dendropsophus counani* Fouquet, Orrico, Ernst, Blanc, Martinez, Vacher, Rodrigues, Ouboter, Jairam & Ron 2015) historically misidentified as *D. parviceps*, was described from the Guiana Shield (Fouquet et al. 2015b). We compared the specimens collected in Serra da Mocidade (Fig. 3h) with known taxa of the *D. parviceps* group, and both morphologic variation and molecular similarity (Fig. 17) reveals their identity as *D. parviceps*. We found a genetic distance of 3% on the 16S fragment used between samples from Serra da Mocidade and the *D. parviceps* sequence available in GenBank, from southwestern Amazonia (Acre state, Brazil), and this phylogenetic divergence can most likely be attributed to intraspecific variation due to wide geographical distance. This species has an extensive distribution throughout Amazonia, including historical records in other mountain ranges of Guayan Highlands (McDiarmid and Paolillo 1988, Schlüter and Mägdefrau 1991), and present high levels of genetic divergence reported between some populations (Fouquet et al. 2015b). It is likely that future studies will reveal other independent lineages hidden under this name, as was the case of *D. counani*.

**Figure 18.**
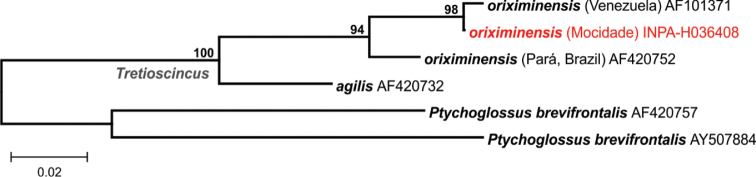
Phylogenetic relationships of *Tretioscincus*. Maximum likelihood phylogenetic tree of *Tretioscincus* species and populations, based on a 427bp fragment of the 16S mtDNA. Only bootstrap values >80% are shown (5,000 replicates). The GenBank accession numbers appear after the names of downloaded sequences, and specimens from the Serra da Mocidade are highlighted.

*Boana boans*, *B. multifasciata*, *B. xerophylla* and *Osteocephalus taurinus* – These medium to large sized arboreal hylids have wide geographical ranges in Amazonia: *B. boans* (Fig. 3i) and *O. taurinus* (Fig. 4b) occur throughout almost the entire basin, *B. xerophylla* (Fig. 3j) occurs on the northern Guiana Shield and *B. multifasciata* (Fig. 4a) in eastern Amazonia and in the transition zone between Amazonia and Cerrado, an open savanna biome with its core distribution in central Brazil (Frost 2017). All these taxa are considered as lowland species, but they may occur in lower densities at higher altitudinal levels, having been recorded on other Guiana Shield highlands mountain ranges (Aubrecht et al. 2012). While *B. boans* and *O. taurinus* are typical of primary and secondary Amazonian forests, *B. xerophylla* and *B. multifasciata* occur in drier habitats, having their evolution intrinsically linked to the development and spread of dry habitats in Brazil. The combination of wide geographical range, presence of allopatric populations with distinct body sizes, colours and calls (Duelmann 1979, Barrio-Amorós and Brewer-Carias 2008), are evidence for a complex of multiple independent lineages (see details on *B. boans* advertisement call from the Serra da Mocidade in Fig. 12, Table 3). Previous studies investigating the intraspecific variation of these taxa have been conducted (e.g., Jungfer et al. 2013, Guarnizo et al. 2016, Orrico et al. 2017), but a clear definition of specific limits still needs further definition.

*Adenomera andreae* and *Leptodactylus petersii* – These species are both terrestrial frogs, typical of Amazonian lowlands (Sá et al. 2014, Frost 2017). The intraspecific molecular variation for *A. andreae*, known to be widely distributed in forest habitats of Amazonia, shows divergent lineages endemic to the Guiana Shield (Fouquet et al. 2014). As for *L. petersii*, several studies have investigated the morphological and bioacoustics variation of the species and their close relatives (*Leptodactylus melanonotus* species group) (Heyer 1970, 1994, de Sá et al. 2014), but the high polymorphism and intraspecific variation of species from this group still hinder definitive taxonomic assignment. Further studies with broader datasets may reveal restricted lineages within *L. petersii*.

*Lithobates palmipes* – This large ranid inhabit forest habitats near to slow-flowing water bodies (lakes, ponds and rivers), is widely distributed in Amazonia, Atlantic Forest and transitional habitats and may represent a species complex. The presence of several gaps in the known species distribution hampers a precise geographical determination (La Marca et al. 2010, Rodrigues et al. 2013). Although the expected occurrence of the species reaches Roraima state and it has already been recorded in nearby areas, no state record has appeared in the literature until this study (nearest documented record at 400 km west of the Serra da Mocidade in Rodrigues et al. 2013). *Lithobates palmipes* abundance also varies considerably across its distributional range (Acosta-Galvis 1999), but the environmental determinants of such variation are still unknown (Ramalho et al. 2011). In the foothills of Serra da Mocidade, the species was abundant in the Pacú River floodplain (Fig. 8b), a tributary of the Branco River, with more than 11 specimens (adults and larvae) recorded in three sampling nights.

*Pseudogonatodes guianensis* – *Pseudogonatodes guianensis* is a small leaf-litter lizard widely distributed within Amazonian lowlands (Ávila-Pires 1995, Ribeiro-Júnior 2015b). However, it is a species apparently rare in the Guiana Shield highlands region, where *Coleodactylus septentrionalis* Vanzolini, 1980 is the more abundant sphaerodactylid (Ribeiro-Júnior 2015b). No record of this species from the Roraima state appears in the previous literature, and our finding filling this distribution gap.

*Plica plica* – This widely distributed arboreal lizard (Ávila-Pires 1995, Rivas et al. 2012) has several distinct lineages with restricted geographical ranges (Murphy and Jowers 2013, Oliveira et al. 2016). However, the genetic divergence between lineages is not clearly reflected in the morphologic variation. A recent study on the morphological variation of the *Plica plica* complex led to the description of several new taxa with restricted distributions and kept *P. plica* as the species with broadest distribution within the Guiana Shield (Murphy and Jowers 2013). Also, a study on the intraspecific genetic variation of this species showed at least two distinct lineages from the region of Serra da Mocidade (Oliveira et al. 2016). We recorded *P. plica* (Fig. 5h) at three altitudinal ranges, and considering such current state of knowledge for the species complex, we retain the specimens from the Serra da Mocidade under this epithet, based in their external morphology.

*Mabuya nigropunctata* – Specimens of *Mabuya* from Serra da Mocidade (Fig. 5g) were assigned to this name based in morphological characters within the known variation of the species, as for example, the paired prefrontal scales, two pairs of nuchal scales, five supraciliar scales, dorsals scales tricarinate, dark ventral surfaces of hand and feet, which are covered by small tubercles, and a dark lateral band, not limited by dorsal and ventral light stripe. This morphological variation promptly differ this specimens from other *Mabuya* with a geographical distribution known for this region: *Mabuya carvalhoi* Rebouças-Spieker and Vanzolini, 1990 (with fused prefrontals, large granules in ventral surface of hands and feets, three to five pairs of nuchals, five longitudinal light stripes along the body and a blue tail) and *Mabuya bistriata* (Spix, 1825) (with four supraciliars, dorsals smooth, one pair of nuchals, distinct light stripes limiting the dark lateral band and at dorsum, and light ventral surfaces of hands, which are covered by moderately large granules). However, the recorded specimens have an interesting characteristic that differs from the species known morphology (Ávila-Pires 1995, Hedges and Conn 2012): the fusion of frontoparietal scales in a single butterfly-shaped scale. Morphological comparisons with other *M. nigropunctata* specimens collected in the Brazilian Guiana Shield highlands region (on Pico da Neblina, deposited at INPA-H collection) reveal the same pattern on the frontoparietal scales for some specimens, while other specimens from the same locality have the typical pattern of two frontoparietals.

Intraspecific analyses of molecular variation indicated a strong genetic structure and multiple lineages within this widely distributed Amazonian taxon (Miralles et al. 2005, Miralles and Carranza 2010). One of those lineages (which have *Mabuya surinamensis* Hallowell, 1857 as available name) occurs on the Guiana and Brazilian Shields, including the Guiana Shield highlands region (Miralles and Carranza 2010). The dissimilarity in the number of frontoparietals found in the specimens from Northern Brazil may represent typical variation within this lineage, but confirmation awaits further taxonomic studies.

*Tretioscincus oriximinensis* – The small cryptic specimens of *Tretioscincus* from Serra da Mocidade (Fig. 5d) were collected on leaf-litter of primary dense forest and have morphological characters within the known diagnosis of *T. oriximinensis*: high number of dorsal scales (>30 rows), polygonal scales on tail, in 12 rows and keeled, prefrontal in contact, six gular scales anteriorly, dorsolateral light stripe become paler at middle of the dorsum (Ávila-Pires 1995). In addition, molecular analyses showed low genetic distance on the 16S fragment used (<0.1%) between Serra da Mocidade specimens and a sample of *T. oriximinensis* from Venezuela. Although the species is apparently more abundant in open habitats, the original description also cites some individuals found in forested areas (Ávila-Pires 1995).

There is a subtle morphological divergence between *T. oriximinensis* populations from northern (including Serra da Mocidade) and eastern Amazonia (Ávila-Pires 1995), with differences in number of ventral scales and extension of keels in scales from tail to posterior dorsals. This morphological divergence is reflected at the molecular level (Fig. 18), as we found ca. 4% of genetic distance between samples from this populations. Dissimilarities between the *T. oriximinensis* populations indicate that their taxonomic status deserves to be further investigated.

*Atractus riveroi* – This groundsnake typical from Guiana Shield highlands was previously known by only two specimens (Roze 1961, Passos et al. 2013). We found three specimens in pitfall traps at the 1,060 m asl altitudinal level: two with a brown dorsal background with dark markings (Fig. 6d) and one with a black dorsal background with small white spots (Fig. 6e) (Passos et al. 2013). For detailed information on morphologic variation and geographical distribution of this species based in these records and new specimens from nearby mountain ranges, see Fraga et al. (2017).

*Chironius fuscus* and *C. septentrionalis* – Two species of diurnal *Chironius* snakes were recorded at Serra da Mocidade: one is a typical lowland species widely distributed in Amazonia and other ecosystems in South America (*Chironius fuscus* – Fig. 6b), while the other is a upland inhabitant (*Chironius septentrionalis* – Fig. 6a, adult male recorded only at 1,060 m asl, with 1,480 mm SVL, 350 mm caudal length – tail damaged, dorsals 12/12/8, ventrals 179, subcaudals 60, anal plate divided, apical pits on neck scales) (Dixon et al. 1993). The latter species was described as a subspecies of the widely distributed Amazonian species *Chironius multiventris* Schmidt and Walker, 1943 (Dixon et al. 1993), but a morphologic taxonomic revision elevated it to specific level (Dixon et al. 1993). This species occurs at high altitudinal levels in adjacent Venezuela, but may occur in lowlands on the island of Trinidad (Dixon et al. 1993). This is the first record from Brazil, extending the known distribution by more than 500 km south.

*Drymobius rhombifer* – Despite being widely distributed in Amazonia (Rivas et al. 2012), this diurnal snake (Fig. 6c) is rarely recorded, apparently due to low densities throughout its range (Stafford and Castro 2010). Our record from Serra da Mocidade is the second known occurrence for the Roraima state, ca. 200 km from the first record (O’Shea and Stimson 1993). Another specimen was collected by one of us (VTC) on the Brazilian Guiana Shield highlands region in a previous expedition to the Neblina mountain range (00°40'N, 65°56'W), in Amazonas state.

*Micrurus remotus* – The holotype of this small monadal coral snake is from the Guiana Shield highlands region [Cerro de la Neblina (Roze 1987)]. Additional specimens have been recorded in this region and in southern Amazonia (Roze 1987, Bernarde et al. 2011, Bernarde and Gomes 2012). Given the great geographical distance and environmental dissimilarity between these two localities, and the overall difficulty in species delimitation due to high intraspecific polymorphism in the species group (Feitosa et al. 2015), a taxonomic revision is required to clarify their status. The specimen from Serra da Mocidade (Fig. 7b) is one of the few documented for the Roraima state (see plate 172 in Campbell and Lamar 2004) and differs slightly in the body colour from patterns described in the literature: it has two light spots on the rostrum, greater extent of white on the head and the white ring after the black nuchal collar is almost imperceptible.

*Platemys platycephala melanonota* – *Platemys platycephala* (Schneider, 1792) is a solitary and nocturnal chelid, which inhabits shallow temporary pools within Amazonian lowland rainforests (Vogt 2008). Several years after their original description, a melanistic colour form was described as a distinct subspecies, possessing a large amount of black pigmentation in the carapace and head and some differences in head scalation in comparison to nominal subspecies (Mendes-Pinto et al. 2012). As this subspecies is more rarely recorded, the total geographical distribution is unknown, but there are records from Peru, Ecuador and Brazil (Mendes-Pinto et al. 2012, van Dijk et al. 2014), which suggest a wide distribution and low abundances throughout the range. The specimen from Serra da Mocidade (Fig. 7g) is the first record of the subspecies in the Roraima state, more than 900 km from the nearest known location (Mendes-Pinto et al. 2012).

## Discussion

Several remarkable herpetofaunal records were found during the first large biological expedition conducted at the Serra da Mocidade mountain range. Based on our results, it is evident that the herpetofauna inhabitant of this mountain range has a greater biotic affinity with lowlands from the Amazon region, but some elements typical of the uplands from Guiana Shield highlands region occurs above 900 m asl.

However, the local species diversity is certainly underestimated in our results, especially at higher altitudinal zones, as this mountain range has an extensive unexplored area of upland forests, which may harbour populations of undescribed amphibians and reptiles typical of the Guiana Shield highlands region. Additionally, the sampling period covered the dry season in the region. This, linked to the fact that the strong El Niño event of 2015-2016 produced higher temperatures throughout the year (Varotsos et al. 2016), may have potentially limited encounter rates of some herpetological groups.

Integrative approaches are increasingly being used in biological inventories (e.g., Vieites et al. 2009, Jansen et al. 2011, Paz and Crawford 2012, Moraes et al. 2016), and are based on several proposed methods (e.g., Padial et al. 2010). The integrative identification approach used here to reveal the species diversity at Serra da Mocidade illustrates the relevance of using different evolutionary data sources to identify taxa from remote and unexplored Amazonian areas. Replication of this method for future Amazonian biodiversity inventories will certainly contribute to a more precise evaluation of species diversity and distribution, as well as the origin, diversification drivers and conservation status of such species.

With the molecular approach, based on reciprocal monophyly, high nodal support and genetic distances of mtDNA, we detected additional samples and extended the distribution of known lineages (*A. apiau*, *R. martyi*, and *T. oriximinensis*, with genetic distances less than 2%), discovered putative divergent lineages of known species (H. aff. taylori, P. aff. vilarsi, *V. ritae*, and *D. parviceps*, with genetic distances between 3–6%), as well as candidate new species (*Stefania* sp., *Epicrionops* sp. and *Brasilotyphlus* sp., with genetic distances between 10–17%). The taxonomic status of the putative new species detected in this study needs to be confirmed using a broader and more detailed analyses of data sources, which may increase the known species diversity at Serra da Mocidade.

Several attempts have been made to define biogeographical sub-regions within Guiana Shield highlands, mainly based on assemblage similarities among isolated mountain ranges (e.g., Mayr and Phelps 1967, Huber 1988a, 1995, McDiarmid and Donnelly 2005). Despite differences in their overall boundaries, all such studies seem to agree on the existence of two main sub-biogeographical regions with high similarity in species composition: the western and eastern Guiana Shield highlands (Mayr and Phelps 1967). This distinction is also observed in the geological origin of regions, as each sub-region is distinct in age and landscape history (Santos et al. 2003). Western Guiana Shield highlands may have experienced a greater faunal exchange during Pleistocene glacial events because the mountains involved lie on a higher basement matrix than Eastern Guiana Shield highlands (Mayr and Phelps 1967, Cook 1974, Cracraft 1985, Kok et al. 2017). At Serra da Mocidade, the herpetofauna composition at highest altitudes was predominantly composed of widespread and altitudinal generalists, with greater similarity to the western sub-region. Occurrence of upland species known to occur in other Guiana Shield highlands mountain ranges (e.g., *A. apiau*, *A. riveroi*) and species closely related to other Guiana Shield highlands endemics (e.g., *Stefania* sp., *Epicrionops* sp.) reinforce the postulated recent high connectivity between highland assemblages of distinct mountain ranges in this region (Kok et al. 2012, Salerno et al. 2012, 2015). Increased gene flow may have occurred during Pleistocene glacial events (Noonan and Gaucher 2005, Lötters et al. 2010, Kok et al. 2012) which made the lowland matrix more permeable, leading to recent events of colonization and speciation. Phylogeographical studies and more extensive sampling are required to address these possibilities more explicitly.

Some species that are often altitudinal-generalists were only found on the Serra da Mocidade at altitudes higher than 900 m (e.g., *D. minutus*, *D. parviceps*, *B. xerophylla*, *A. punctatus*, *B. b. bilineatus*). The Serra da Mocidade mountain range has a complex hydrologic mosaic, with streams that vary in their basin origins and amounts of dissolved sediments (Barbosa 2005, Ministério do Meio Ambiente 2016), which generates high habitat heterogeneity. The absence of some altitudinal generalists at lower altitudes (<900 m asl) may be related to a balance between physiological tolerance and habitat conditions, or to sampling bias. Long-term sampling would be necessary to fully elucidate species distribution patterns along these altitudinal gradients.

The results from this short-term multidisciplinary expedition (see other scientific results in Bastos et al. 2016, Neto et al. 2016, Dantas and Hamada 2017, Fraga et al. 2017, Lourenço 2017, Raimundi et al. 2017, and the documentary associated with the expedition at http://www.grifafilmes.com/en/new-species) highlight our poor knowledge of the Brazilian Guiana Shield highlands region. Our study can be used as primary data source for future biodiversity, biogeography, and conservation assessments that consider Guiana Shield highlands on a larger scale. We encourage additional initiatives to enable costly and logistically difficult expeditions to remote Amazonian areas, to fill-in basic knowledge gaps of biological diversity in remote areas, and investigate the processes that led to the currently observed biodiversity patterns.

## Supplementary materials

Supplementary material 1 Specimens examined Data type: (measurement/occurence/multimedia/etc.) This dataset is made available under the Open Database License (http://opendatacommons.org/licenses/odbl/1.0/). The Open Database License (ODbL) is a license agreement intended to allow users to freely share, modify, and use this Dataset while maintaining this same freedom for others, provided that the original source and author(s) are credited.Leandro J.C.L. Moraes, Alexandre P. de Almeida, Rafael de Fraga, Rommel R. Rojas, Renata M. Pirani, Ariane A.A. Silva, Vinícius T. de Carvalho, Marcelo Gordo, Fernanda P. Werneck
