# Supplementary material for: Integrative overview of the herpetofauna from Serra da Mocidade, a granitic mountain range in northern Brazil
Source: Zookeys. 2017 Nov 22;(715):103–59. doi: 10.3897/zookeys.715.20288 (PMC5740401; doi:10.3897/zookeys.715.20288)
Supplement: Supplementary material 1 — Specimens examined [file zookeys-715-103-s001.docx]

**Supplementary file 1. Accession numbers of specimens examined.**

**Brazil, Roraima, Serra da Mocidade.** **Amphibia:** *Adenomera andreae* (INPA-H036438)**;** *Allophryne ruthveni* (INPA-H036363); *Anomaloglossus apiau* (INPA-H035355, 036279, 036282, 036283, 036289, 036293, 036306, 036308, 036310, 036311, 036317-22, 036327, 036328, 036338-40, 036347, 036348, 036350, 036353, 036354, 036356, 036357, 036359, 036364, 036371, 036386, 036390, 036396-99, 036411, 036412, 036414, 036415, 036434-37, 036439-42, 036454, 036455); *Boana boans* (INPA-H036231, 036233, 036240, 036261, 036265, 036277, 036287, 036329, 036370, 036447, 036458-61, 036475-77, 036486); *Boana multifasciata* (INPA-H036276, 036335, 036352); *Boana xerophylla* (INPA-H036288); *Brasilotyphlus* sp. (INPA-H036309, 036457); *Dendropsophus minutus* (INPA-H035401, 036387, 036388, 036402, 036403, 036418, 036430, 036431, 036443-46, 036448-50, 036452, 036453); *Dendropsophus parviceps* (INPA-H036290, 036292, 036304, 036305, 036312, 036365); *Epicrionops* sp. (INPA-H036291); *Hyalinobatrachium* aff. *taylori* (INPA-H036280, 036284, 036299, 036301, 036362); *Leptodactylus guianensis* (INPA-H036479, 036487); *Leptodactylus mystaceus* (INPA-H036245, 036404, 036424-26, 036433); *Leptodactylus petersii* (INPA-H036273); *Lithobates palmipes* (INPA-H036470-74, 036482-85, 036491); *Osteocephalus taurinus* (INPA-H036232. 036234-39, 036241-43, 036250-52, 036267, 036282, 036323, 036324, 036337); *Physalaemus ephippifer* (INPA-H036266, 036268, 036274, 036275, 036325, 036334); *Pristimantis* aff. *vilarsi* (INPA-H036246, 036262-64, 036281, 036285, 036286, 036303, 036313-16, 036326, 036330-33, 036341-46, 036351, 036360, 036361, 036366, 036367, 036372); *Rhaebo guttatus* (INPA-H036271, 036468, 036480, 036496-98); *Rhinella marina* (INPA-H036256-58, 036270, 036272); *Rhinella martyi* (INPA-H035260, 035369, 036247-49, 036253-55, 036269, 036278, 036307, 036336, 036368); *Stefania* sp. (INPA-H036419, 036391); *Vitreorana ritae* (INPA-H036294-98, 036300, 036302, 036349, 036358). **Reptilia:** *Ameiva ameiva ameiva* (INPA-H036377, 036409, 036413); *Anolis fuscoauratus* (INPA-H036373, 036378-80, 036384, 036394, 036395, 036405, 036407, 036427-29); *Anolis punctatus* (INPA-H035417) *Anolis planiceps* (INPA-H036385); *Atractus riveroi* (INPA-H036422, 036432, 036451); *Bothrops atrox* (INPA-H036467, 036500); *Bothrops bilineatus bilineatus* (INPA-H036420, 036421); *Cercosaura ocellata* (INPA-H036383); *Chironius fuscus* (INPA-H036465, 036494); *Chironius septentrionalis* (INPA-H036499); *Dipsas catesbyi* (INPA-H036381); *Dipsas indica indica* (INPA-H036463); *Dipsas pavonina* (INPA-H036786); *Drymobius rhombifer* (INPA-H036469); *Imantodes cenchoa* (INPA-H036464); *Lachesis muta muta* (INPA-H036622); *Mabuya nigropunctata* (INPA-H036375, 036376, 036410, 036423); *Micrurus remotus* (INPA-H036389); *Paleosuchus trigonatus* (INPA-H036481); *Plica plica* (INPA-H036374, 036392, 036393, 036492, 036493, 036495); *Polychrus marmoratus* (INPA-H036466); *Pseudogonatodes guianensis* (INPA-H036400, 036416, 036456); *Thecadactylus rapicauda* (INPA-H036478, 036488-90); *Tretioscincus oriximinensis* (INPA-H036406, 036408); *Xenodon rabdocephalus rabdocephalus* (INPA-H036462).
